# Cellular Pushing Forces during Mitosis Drive Mitotic Elongation in Collagen Gels

**DOI:** 10.1002/advs.202000403

**Published:** 2021-01-04

**Authors:** Sungmin Nam, Yung‐Hao Lin, Taeyoon Kim, Ovijit Chaudhuri

**Affiliations:** ^1^ Department of Mechanical Engineering Stanford University 418 Panama Mall Stanford CA 94305 USA; ^2^ John A. Paulson School of Engineering and Applied Sciences Wyss Institute for Biologically Inspired Engineering Harvard University 58 Oxford Cambridge MA 02138 USA; ^3^ Department of Chemical Engineering Stanford University 418 Panama Mall Stanford CA 94305 USA; ^4^ Weldon School of Biomedical Engineering Purdue University 206 S Martin Jischke Drive West Lafayette IN 47907 USA

**Keywords:** biophysics, cell division, collagen gels, cytokinesis, extracellular matrix, mechanotransduction, tumor growth

## Abstract

Cell elongation along the division axis, or mitotic elongation, mediates proper segregation of chromosomes and other intracellular materials, and is required for completion of cell division. In three‐dimensionally confining extracellular matrices, such as dense collagen gels, dividing cells must generate space to allow mitotic elongation to occur. In principle, cells can generate space for mitotic elongation during cell spreading, prior to mitosis, or via extracellular force generation or matrix degradation during mitosis. However, the processes by which cells drive mitotic elongation in collagen‐rich extracellular matrices remains unclear. Here, it is shown that single cancer cells generate substantial pushing forces on the surrounding collagen extracellular matrix to drive cell division in confining collagen gels and allow mitotic elongation to proceed. Neither cell spreading, prior to mitosis, nor matrix degradation, during spreading or mitotic elongation, are found to be required for mitotic elongation. Mechanistically, laser ablation studies, pharmacological inhibition studies, and computational modeling establish that pushing forces generated during mitosis in collagen gels arise from a combination of interpolar spindle elongation and cytokinetic ring contraction. These results reveal a fundamental mechanism mediating cell division in confining extracellular matrices, providing insight into how tumor cells are able to proliferate in dense collagen‐rich tissues.

## Graphical abstract

Single cells cultured in 3D confining collagen gels generate substantial pushing forces on the surrounding matrix to drive mitotic elongation, a critical aspect of cell division. Neither cell spreading prior to mitosis, nor matrix degradation during spreading or mitotic elongation, is required. Mechanistically, the pushing forces arise from a combination of interpolar spindle elongation and cytokinetic ring contraction.

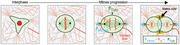

## 1 Introduction

**Figure 1:**
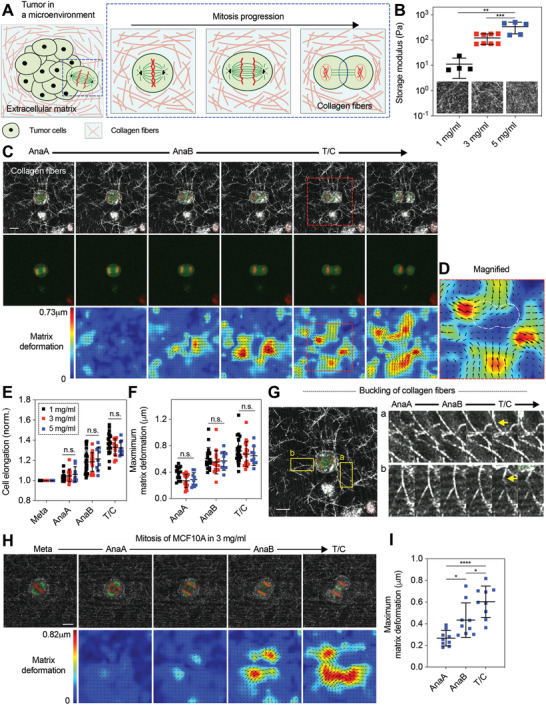
Cells dividing in collagen gels generate extracellular pushing forces along the mitotic axis that deform the surrounding collagen fibers from anaphase to telophase/cytokinesis. A) Schematic of a cell dividing in a collagen‐rich stromal microenvironment. B) Shear storage moduli of collagen gels at the indicated densities. Inset, reflectance images of collagen networks with varying density. C) Fluorescence images of a dividing cell along with reflectance images of collagen fibers (top row), the dividing cell alone (middle row), and corresponding matrix displacement maps (bottom row) overlaid with displacement vectors (black). 1 mg mL^−1^ collagen gels were used. See Video S1 in the Supporting Information. Red dotted box indicates region of interest to be magnified in (D). Here and in all other figures, red and green indicate histones labeled with red fluorescent protein (RFP) and microtubules labeled with green fluorescent protein (GFP), respectively. D) Magnified image of the region indicated in (C). E) Quantification of cell elongation in collagen gels of varying density at each mitotic stage (*n* = 9–22, *N* > 3). The length of cell body was normalized by the initial length. F) Assessment of maximum matrix deformation generated during division in collagen gels of varying density (*n* = 9–22, *N* > 3). G) Buckling of collagen fibers were observed along mitotic axis during elongation (yellow arrow), indicating pushing force generation. See Video S2 in the Supporting Information. Out of 31 mitotic cells, 10 cells were found to buckle one or two collagen fibers surrounding the cells during division (≈32%). H) Fluorescence images of a dividing MCF10A cell along with reflectance images of collagen fibers (top row), and corresponding matrix displacement maps (bottom row) overlaid with displacement vectors (black). 3 mg mL^−1^ collagen gels were used. I) Assessment of maximum matrix deformation generated by MCF10A cells during division in collagen gels (*n* = 9, *N* > 3). B,E,F,I) One‐way analysis of variance with Tukey's multiple comparison; n.s. not significant between all groups. ^*^ *p* < 0.05, ^**^ *p* < 0.01, ^***^ *p* < 0.001, and ^****^ *p* < 0.0001. Data are presented as mean ± SD. Scale bars, 10 µm.

Unregulated cell division drives the growth of primary and metastatic tumors. Many solid tumors typically grow in dense, and sometimes stiffened, stromal tissues that would be expected to resist tumor growth mechanically.^[^ ^1^, ^2^ ^]^ Stromal tissues typically consists of type‐1 collagen rich extracellular matrix (ECM) as well as stromal cells.^[^ ^3^, ^4^, ^5^ ^]^ Cell division involves numerous morphological changes including cell growth and mitotic elongation, which is important for proper segregation of chromosomes and intracellular materials.^[^ ^6^, ^7^ ^]^ We previously found that mitotic elongation is a key requirement for cell division, with mitosis not progressing beyond metaphase when mitotic elongation is blocked.^[^ ^8^ ^]^ Thus, for tumor growth to occur, tumor cells must overcome the mechanical constraints imposed by the surrounding microenvironment to undergo mitotic elongation (**Figure** **1**A). Indeed, most solid tumors are under mechanical compression.^[^ ^5^ ^]^ However, the mechanisms that drive mitotic elongation in collagen‐rich matrices remain unclear.

In principle, several possible mechanisms could facilitate mitotic elongation of cancer cells in collagen‐rich matrices. Gels of reconstituted type‐1 collagen have been widely used as in vitro scaffolds that provide a microenvironment closely mimicking collagen‐rich stromal tissues.^[^ ^9^, ^10^, ^11^ ^]^ It is known that cells can spread in type‐1 collagen gels through mechanical force and protease‐mediated degradation.^[^ ^12^, ^13^ ^]^ Due to the mechanical plasticity of type‐1 collagen gels, deformation of the matrices generated by physical forces of cells remains permanent and provides additional space to the spreading cells.^[^ ^14^, ^15^, ^16^ ^]^ Studies investigating division of spreading cells in 3D matrices have found that the axis of cell division is directed by the axis of cell spreading,^[^ ^17^, ^18^ ^]^ and that cells divide into matrix voids created by the cell spreading,^[^ ^17^ ^]^ suggesting a role of cell spreading in cell division. However, it remains unclear whether space created during cell spreading is sufficient for mitotic elongation. In addition to physical forces, cells are also known to use matrix metalloproteinases (MMPs), enzymes that biochemically degrade matrices, facilitating matrix remodeling and creation of space. For example, cancer cells are thought to use MMPs to cleave adjacent collagen fibers and clear the structural barriers, when they infiltrate other tissues.^[^ ^13^, ^19^, ^20^, ^21^, ^22^ ^]^ Therefore, cells could utilize matrix degradation to clear space for mitotic elongation either during cell spreading, or during mitosis itself. A final possibility comes from our recent finding that cells are able to generate protrusive extracellular forces during mitosis to create space for mitotic elongation in alginate hydrogels.^[^ ^8^ ^]^ Alginate hydrogels are bio‐inert scaffolds that are not susceptible to degradation by MMPs and, in the previous study, did not present cell‐adhesion binding motifs so that integrin or protease‐mediated matrix remodeling were not possible. Forces were generated through interpolar spindle elongation, which couples to the hydrogel via the astral microtubules, and cytokinetic ring contraction, which drives expansion along the mitotic axis due to volume conservation. However, the mechanisms that cancer cells utilize to drive mitotic elongation in confining type‐1 collagen matrices, where integrin and protease‐mediated remodeling are possible, remain unclear.

Here we investigated division of cancer cells cultured in collagen gels that mimic collagen‐rich stromal microenvironments. We found that mitotic cells generate protrusive forces that physically deform the surrounding collagen fibers to allow for mitotic elongation. Mitotic extracellular force generation was similar between rounded cells and cells that were first allowed to spread and remodel the surrounding matrix prior to mitosis, as well as cells dividing while proteases were inhibited. Mechanistically, elongation of interpolar spindles and lateral contraction by cytokinetic ring, the two sources identified in our previous study, drive protrusive force generation during cell division in collagen gels. Together, these findings establish that cancer cells utilize protrusive extracellular force generation during mitosis, and not protease degradation or spreading, to drive mitotic elongation in type‐1 collagen gels.

## 2 Results

### 2.1 Cell‐Generated Pushing Forces Drive Mitotic Elongation in Collagen Gels

To determine how mitotic cells divide in collagen‐rich microenvironments, we studied the division of MDA‐MB‐231 breast cancer cells cultured in collagen gels. Cells were stably transfected with green fluorescent protein (GFP)‐labeled *α*‐tubulin and red fluorescent protein (RFP)‐labeled histone to allow visualization of the mitotic spindle and determination of the mitotic phases during live‐cell imaging.^[^ ^8^ ^]^ Varying densities of collagen gels of 1 mg mL^−1^ (low), 3 mg mL^−1^ (medium), and 5 mg mL^−1^ (high) were used in order to provide different collagen‐rich microenvironments to the dividing cells. The elastic modulus of the collagen gels was measured to increase from ≈10 to ≈400 Pa with increasing density (Figure 1B). This spans the range of densities typically used to mimic stromal matrices in vitro,^[^ ^23^ ^]^ and the elastic moduli of the collagen gels overlap with the lower range of elastic moduli reported in soft tissues.^[^ ^10^, ^24^, ^25^ ^]^ Creep‐recovery tests demonstrated that mechanical plasticity was similar across these densities for creep times of 5 min, a time‐scale relevant to mitosis (Figure S1, Supporting Information). As shown in previous studies,^[^ ^14^, ^26^ ^]^ low‐density collagen gels formed networks with longer and more distinct fibers, whereas higher‐density collagen gels exhibited a finer meshed structure with shorter and indistinct fibers (Figure 1B, inset).

First, we probed whether dividing cells are able to generate protrusive extracellular forces along the mitotic axis during mitosis in collagen gels in the absence of cell spreading. Prior to encapsulation in the low‐density collagen gels, cells were synchronized at prometaphase using thymidine and S‐trityl‐l‐cysteine (STLC), cell cycle inhibitors, and then released from the inhibitors while encapsulated as single cells in the collagen gels. This approach allows cells to undergo mitosis shortly after encapsulation, so that the cells did not have an opportunity to spread in the gels. While proceeding through mitosis in the low‐density gels, cells were found to deform the surrounding collagen fibers in a protrusive manner along the mitotic axis (Figure 1C–E; Video S1, Supporting Information). Matrix deformation of collagen gels due to force generation increased throughout mitosis, with the matrix deformation at anaphase B (AnaB) and telophase/cytokinesis (T/C) being the greatest (Figure 1F). Interestingly, some collagen fibers located in the direction of the mitotic axis that were aligned along this axis, buckled at AnaB and T/C (Figure 1G; Figure S2 and Video S2, Supporting Information). Since buckling of collagen fibers occurs when compressive forces on the fibers reach a critical value, this observation directly confirmed that mitotic cells exert protrusive extracellular forces on the collagen fibers along the mitotic axis. To confirm that synchronization with STLC did not alter force generation during mitosis, a cdk1 inhibitor, RO3306, was also used for synchronization. RO3306‐treated cells did not show any significant change in cell elongation and spindle elongation during mitosis, and generated matrix deformation to the extent similar to the cells with STLC, confirming the result (Figure S3, Supporting Information). In medium and high‐density gels, cells were also observed to deform the surrounding collagen fibers along the mitotic axis to levels similar to those in the low‐density gels (Figure 1F; Figure S4 and Videos S3 and S4, Supporting Information). As collagen gels of higher density exhibit higher stiffness, these results indicate that cells are able to generate higher forces in the higher density of gels in order to undergo mitotic elongation properly. In addition, it was observed that collagen fibers along the axis perpendicular to the mitotic axis moved inwardly across all the densities, as cells progressed mitosis (Figure 1E; Figure S4, Supporting Information). A recent study examining division of zebrafish epicardial cells in monolayers found a direct connection between dividing cells and the underlying substrate, which leads to inward movement of the substrate along the perpendicular axis as cells divide.^[^ ^27^ ^]^ However, in our study, clear matrix voids were observed between the cytokinetic ring of dividing cells and the surrounding matrices (Figure S5, Supporting Information). This indicates that cytokinetic ring contraction does not drive this inward matrix deformation, suggesting that extracellular force generation along the mitotic axis instead may be the underlying driver. Finally, to further establish the physiological relevance of force generation during mitosis to noncancer cells, we performed experiments with MCF10A cells, often used as a model of normal mammary epithelium. To visualized chromatids and microtubules, cells were labeled with RFP‐histone and SiR‐tubulin dye, respectively. Cells were then synchronized, encapsulated into the medium‐density gels, and observed for mitosis. Similar to MDA‐MB‐231 cells, MCF10A cells were also found to deform the surrounding collagen fibers along the mitotic axis, with matrix deformation being the greatest at AnaB and T/C (Figure 1H,I). This indicates that extracellular force generation during mitosis is also relevant to normal cells, and not just cancer cells.

### 2.2 Spread Cells Undergoing Mitosis Also Generate Pushing Forces for Mitotic Elongation

**Figure 2:**
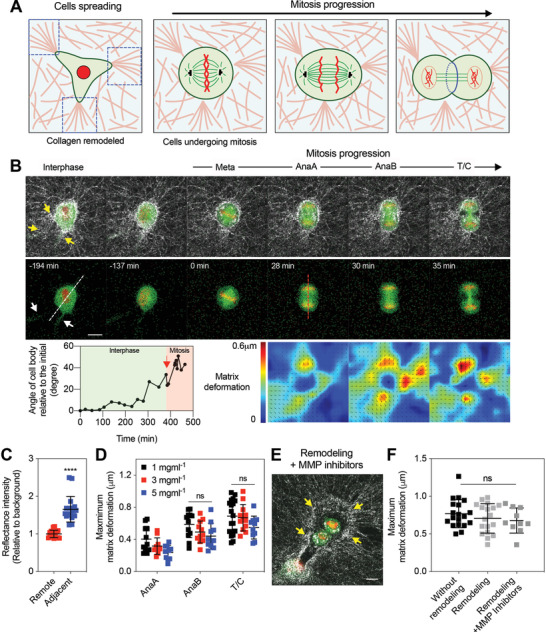
Matrix remodeling and proteolytic degradation do not diminish matrix deformation generated during mitotic elongation. A) A schematic of a cell undergoing mitosis after remodeling the surrounding collagen network. B) Fluorescence images of a cell entering mitosis from interphase along with reflectance images of collagen fibers (top row), the cell alone (middle row), and corresponding matrix displacement maps (bottom row). The yellow and white arrows indicate densified collagen fibers and the protrusion of the cell body, respectively. The white and red dotted lines indicate the long axis of the cell body at the interphase and the mitotic axis during mitosis, respectively. The graph represents the angle of cell body axis as a function of time. The red arrow indicates when the cell enters metaphase. The indicated experiments were conducted in 3 mg mL^−1^ collagen gels. C) Reflectance intensity of collagen fibers at regions adjacent to cells, or within 20 mm, and at regions remote from cells, or greater than 20 mm (*n* = 18, *N* > 3). Student's *t*‐tests were used; ^****^ *p* < 0.0001. D) Maximum matrix deformation associated with each mitotic stage (*n* = 10–18, *N* > 3). E) Fluorescence images of a dividing cell treated with a cocktail of MMP inhibitors. Collagen fibers were represented with white color. 1 mg mL^−1^ collagen gels were used. F) Maximum matrix deformation generated by cells that divided shortly after encapsulation (without remodeling), cells undergoing division after one‐day culture (remodeling), and cells treated with MMP inhibitors (*n* = 10–19, *N* > 3). D,F) One‐way analysis of variance with Tukey's multiple comparison; n.s. not significant between all groups. Data are presented as mean ± SD. Scale bars, 10 µm.

Next, we examined whether cells that were allowed to spread and remodel the surrounding matrix prior to mitosis also generate protrusive extracellular forces during mitosis (**Figure** **2**A). For this set of experiments, cells were encapsulated in collagen gels and cultured for one day while being synchronized at mitosis. During the one‐day culture in collagen gels, cells were expected to spread and mechanically remodel the surrounding collagen network. Indeed, substantial morphological differences were observed between cells with and without the one‐day culture, with cells spreading after one day of culture as indicated by cell circularity and spreading area (Figure S6A–D, Supporting Information). In addition, densified collagen fibers were found around cells with one‐day culture, and the density of collagen fibers, indicated by reflectance intensity, near the cells was measured to be significantly higher than that far from the cells (Figure 2B,C; Figure S6E, Supporting Information). In contrast, for cells without the one‐day culture, there was no significant difference in reflectance intensity of collagen fibers between the vicinity of the cells and further away (Figure S7A, Supporting Information). Therefore, the observed densification of collagen fibers around cells with the culture was attributed to matrix remodeling and cell spreading, which occurred during the 24 h culture period. Next, matrix deformation during division of the spread cells was examined. Notably, spread cells undergoing mitosis after reorganizing the surrounding collagen network pushed away the surrounding collagen fibers along the mitotic axis, similar to the cells that divided without matrix remodeling (Figure 2B; Video S5, Supporting Information). Matrix deformation was observed to increase throughout mitosis, with the greatest deformation occurring at AnaB and T/C (Figure 2D; Figure S6E, Supporting Information). Interestingly, the axis of cell division was found to be different from the long cell axis at interphase in some cases (Figure 2B), indicating that the division axis is not predetermined during interphase. In addition to the low‐density gels, we also examined mitosis of spread cells in the medium and high‐density gels. Spread cells in these gels were also observed to deform the surrounding collagen fibers along the mitotic axis to the extent similar to those in the low‐density gels (Figure 2D). These results indicate that collagen remodeling during cell spreading is not sufficient to create space for mitotic elongation, and therefore cells still require extracellular pushing force generation to complete division.

### 2.3 Proteolytic Degradation is not Necessary for Mitotic Elongation

After finding that cell spreading does not generate sufficient space for mitotic elongation, we next explicitly examined the role of MMP activity in cell division, as matrix degradation could contribute to mitotic elongation during mitosis itself. Cells were encapsulated in collagen gels and cultured for one day as in the spread cell experiments. To broadly inhibit the MMP activity, a cocktail of protease inhibitors, including marimastat, E‐64, aprotinin, and leupeptin, was added to the medium. This combination of inhibitors has been shown to effectively block the activity of MMPs.^[^ ^28^ ^]^ However, densified collagen fibers were still observed around the cells treated with the inhibitors at a level similar to that observed around untreated cells, suggesting only a minimal role of proteolytic degradation in spreading under the conditions used in this study (Figure 2E; Figure S7B, Supporting Information). This is consistent with previous studies showing that cells are able to mechanically remodel collagen networks using physical forces without the aid of biochemical degradation.^[^ ^14^, ^15^, ^29^ ^]^ Further, inhibition of MMPs did not perturb progression of mitosis, and importantly, matrix deformation by the treated cells occurred at levels similar to that of cells without the MMP inhibition (Figure 2F; Figure S7C and Video S6, Supporting Information). These results demonstrate that proteolytic degradation is not required for mitotic elongation in collagen matrices.

### 2.4 Spindle Elongation and Cytokinetic Ring Contraction Drive Pushing Forces for Mitotic Elongation

**Figure 3:**
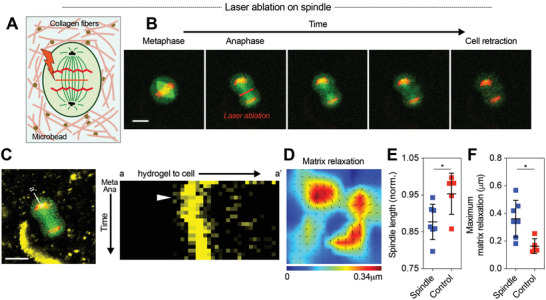
Protrusive force generation arises in part from interpolar spindle elongation. A) A schematic of laser ablation experiments. Fluorescent microbeads embedded in collagen gels enabled visualization of matrix relaxation due to laser ablation. B) Fluorescence images of a cells undergoing laser ablation of interpolar spindle at anaphase. 3 mg mL^−1^ collagen gels were used. See Video S7 in the Supporting Information. C) A kymograph of microbead movement before and after ablation. The line tracked over mitosis is indicated (left). The time scale of the kymograph is ≈2 s. The white arrow indicates when laser ablation was performed. D) Matrix relaxation field associated with laser ablation experiment. E) Spindle length and F) maximum matrix relaxation after ablation for mitotic spindle (spindle) and for a region where spindle was not located (control) (*n* = 5–7, *N* > 3). Data are presented as mean ± SD. Student's *t*‐tests were used; ^*^ *p* < 0.05. Scale bars, 10 µm.

After establishing that cellular pushing forces generate space for mitotic elongation of dividing cells in collagen gels, we investigated the underlying biophysical mechanisms. We previously found that cellular pushing forces during mitosis in alginate gels resulted from interpolar spindle elongation and lateral contraction by the cytokinetic ring, which drives mitotic expansion due to volume conservation.^[^ ^8^ ^]^ Therefore, we tested whether these previously identified mechanisms also underlie force generation in collagen gels. First, mitosis of cells dividing without spreading was examined. To directly investigate the role of interpolar spindle elongation in pushing force generation, laser ablation experiments were performed. Fluorescent microbeads with an average size of 1.5 µm were embedded in collagen gels to clearly visualize matrix deformation, and the medium density collagen gels were used so that the microbeads were firmly embedded in the network (Figure S8, Supporting Information).^[^ ^30^, ^31^ ^]^ Cells at prometaphase were encapsulated in the gels, and were allowed to proceed through mitosis. Once cells entered anaphase, mitotic spindles were severed using laser ablation and the displacement of beads embedded in the collagen gels was traced before and after ablation (**Figure** **3**A). Following ablation, elongation of mitotic spindles was relaxed, and microbeads located on the mitotic axis immediately displaced in the direction of the cells after spindle ablation, indicating the relaxation of pushing forces from the cell (Figure 3B,C; Video S7, Supporting Information). More broadly, bead tracking analysis indicated that the accumulated deformation due to protrusive forces during mitosis was partially relaxed (Figure 3D). To rule out the possibility that the relaxation of deformation arose from cell death due to laser exposure or from collapse of other intracellular structures, laser ablation was performed on part of cell body where spindles were not positioned along an axis parallel to the interpolar spindle (Figure S9A and Video S8, Supporting Information). Contrary to the ablation on spindles, laser ablation performed on nonspindle regions did not disrupt spindle elongation (Figure 3E). In addition, microbeads around the cells subjected to the ablation remained in their original positions, and matrix relaxation did not occur. (Figure 3F; Figure S9B,C, Supporting Information). The partial relaxation of matrix deformation due to ablation of interpolar spindles indicates that elongation of the interpolar spindle is at least partially responsible for the force generation that deforms the surrounding collagen matrix during mitosis.

In addition to the laser ablation experiments, pharmacological perturbation of interpolar spindles was performed to confirm the role of the interpolar spindle in generating pushing forces. STLC was introduced to mitotic cells at a low concentration to partially inhibit kinesin‐5 activity, while not blocking mitotic progression.^[^ ^32^, ^33^ ^]^ The partial inhibition of kinesin‐5 activity was found to decrease matrix deformation during mitosis, which is consistent with our previous results (Figure S10A,B, Supporting Information).^[^ ^8^ ^]^ Together, these results demonstrate the role of interpolar spindle elongation in generating pushing forces during mitosis in collagen gels.

**Figure 4:**
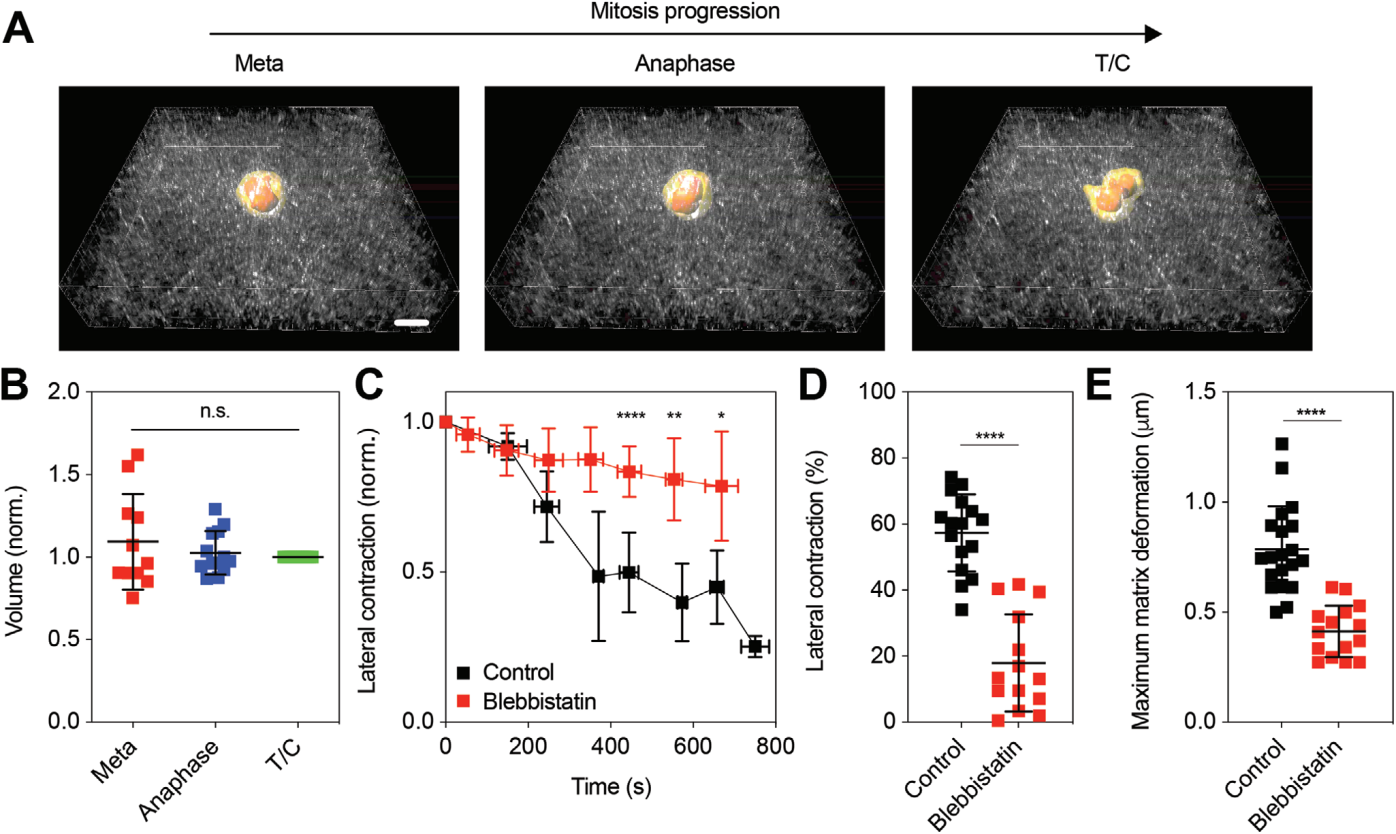
Lateral contraction by cytokinetic ring also contributes to protrusive force generation. A) Three‐dimensionally reconstructed images of dividing cells at the corresponding mitotic stages for cells cultured in 1 mg mL^−1^ collagen gels. See Video S9 in the Supporting Information. B) Total volumes of cells over mitosis. Cell volumes were normalized by the value at T/C (*n* = 11–13, *N* > 3). One‐way analysis of variance with Tukey's multiple comparison; n.s. not significant between all groups. C) Change in width of dividing cells treated with blebbistatin and without treatment (control), over time. The width was normalized by the initial value. Student's *t*‐tests were used to compare lateral contraction between groups at selected time points; ^*^ *p* < 0.05, ^**^ *p* < 0.01, and ^****^ *p* < 0.0001. D) Comparison of lateral contraction from the initial width for treated cells (blebbistatin) and nontreated cells (control) (*n* = 14–16, *N* > 3). Student's *t*‐tests were used; ^****^ *p* < 0.0001. E) Comparison of maximum matrix deformation generated by treated cells (blebbistatin) and nontreated cells (control) (*n* = 15–20, *N* > 3). Student's *t*‐tests were used; ^****^ *p* < 0.0001. Data are presented as mean ± SD. Scale bars, 10 µm.

After confirming the contribution of interpolar spindle elongation to cellular pushing forces in collagen gels, we investigated whether lateral contraction driven by cytokinetic ring also contributes. The volume of cells during mitosis was found to be conserved over mitosis (**Figure** **4**A,B; Video S9, Supporting Information). Since the volume is conserved, contraction of a mitotic cell body at its equator, driven by cytokinetic actomyosin ring contraction, would be expected to displace intracellular materials to the poles, resulting in outward expansion along the mitotic axis and generation of pushing forces.^[^ ^8^ ^]^ To validate the role of cytokinetic ring contraction in force generation, we introduced blebbistatin, a potent inhibitor of myosin II, to dividing cells to inhibit cytokinetic ring contraction. Inhibition of cytokinetic ring contraction with blebbistatin substantially reduced lateral contraction of mitotic cells (Figure 4C,D; Figure S10C, Supporting Information). Importantly, deformation of collagen network by the treated cells was significantly decreased compared to nontreated cells (Figure 4E). These results indicate that contraction of the cytokinetic ring also contributes to pushing forces generated during mitosis.

Next, we examined whether these mechanisms also underlie force generation during mitosis of spread cells. Similar to the pharmacological inhibition experiments on cells dividing without spreading, STLC at a low concentration and blebbistatin were respectively added to cells undergoing mitosis after spreading and matrix remodeling. Both the partial inhibition of kinesin‐5 and inhibition of actomyosin were shown to decrease matrix deformation during mitosis (Figure S11, Supporting Information), confirming that interpolar spindle elongation and cytokinetic ring contraction are also involved in generating pushing forces in mitotic cells that spread and remodel matrix prior to mitosis.

**Figure 5:**
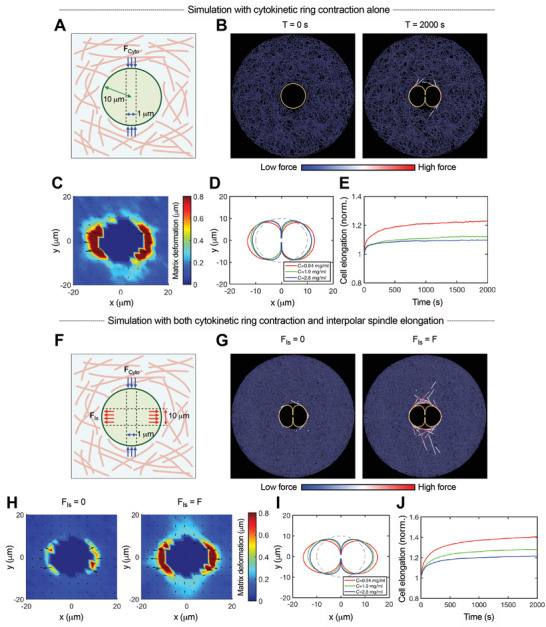
Computational simulations indicate contributions of both cytokinetic ring contraction and interpolar spindle elongation to protrusive extracellular force generation along the mitotic axis. A) A schematic depicting a model of cell division in a collagen gel, in which a constant inward force was applied to the cell membrane near the cell equator to simulate cytokinetic ring contraction. B) Snapshots of simulations at the initial (*T* = 0 s) and final time (*T* = 2000 s). Orange and blue colors represent the cell membrane and matrix fibers, respectively. Some of the fibers with high tensile forces are highlighted via color scaling. C) Matrix deformation led by cytokinetic ring contraction at the final time (*T* = 2000 s). Fiber concentration in (B,C) is estimated to be 0.94 mg mL^−1^. D) Change in cell shape under varying fiber concentration. E) Cell elongation as a function of time for the simulations. The instantaneous length of a cell was normalized by the initial length. F) A schematic depicting a model incorporating both cytokinetic ring contraction and spindle elongation. A constant outward force was added to the cell membrane in the direction of the mitotic axis in order to simulate the contribution of spindle elongation. G) Snapshots of simulations with and without incorporating the outward force. H) Matrix deformation corresponding to cases shown in (G). Fiber concentration in (G,H) is estimated to be 2.8 mg mL^−1^. Note that when cell elongation is facilitated due to spindle elongation, the magnitude of inward flow of matrix along the perpendicular axis increases. I) Change in cell shape under varying fiber concentration. J) Cell elongation as a function of time. The instantaneous length of a cell was normalized by the initial length.

To gain more insight into the role of the cytokinetic ring in generating pushing forces along the mitotic axis, we performed computational simulations of a cell dividing in a collagen matrix (**Figure** **5**A). First, fiber networks that captured the shear modulus, nonlinear elasticity, and strain‐enhanced stress relaxation of collagen gels were generated computationally^[^ ^15^, ^26^ ^]^ (Figure S12, Supporting Information; see the Experimental Section). Then, the division of a cell inside this network was modeled. To simulate contraction of the cytokinetic ring, a constant inward force was applied to the cell membrane at the cell equator, and changes in cell shape were observed. A constraint of constant cell volume during division was applied in order to be consistent with the experimental observation. While lateral contraction proceeded, the cell body expanded along the mitotic axis due to bending stiffness of the cell membrane and volume conservation, thereby pushing out the surrounding fibers along the mitotic axis (Figure 5B,C; Figure S13, Supporting Information). The degree of the cell expansion decreased as the fiber density increased, with a maximum elongation of around 20% in the low‐density matrix (Figure 5D,E). The observed maximum elongation of 40% in 1 mg mL^−1^ collagen gels from experiments indicates that cytokinetic ring contraction alone does not account for all the force generation. Nonetheless, this model confirms that contraction of cytokinetic ring alone contributes to generation of pushing forces along the mitotic axis.

Next, we used the model to explore the contribution of interpolar spindle elongation to force generation. Since elongation of interpolar spindles directly leads to outward force generation, a constant outward force was added along the cell membrane in the direction of the mitotic axis (Figure 5F). Incorporation of the outward force facilitated elongation of the cell body and increased deformation of the surrounding fibers (Figure 5G,H; Figure S13, Supporting Information). With the contribution of interpolar spindle elongation, overall cell elongation obtained from the simulations with 0.94 mg mL^−1^ approached levels similar to those observed in the experiments with 1 mg mL^−1^, further supporting the role of spindle elongation in generating protrusive forces (Figures  and 5I,J). In addition, when spindle elongation facilitated cell elongation and thereby further deformed the surrounding matrix, substantial inward movement of fibers along the perpendicular axis was observed (Figure 5H; Figure S13C–E, Supporting Information). This was consistent with the inward movement of a matrix observed in the experiments (Figure 1D; Figure S4, Supporting Information) and validated the idea that the matrix being pushed out along the mitotic axis due to protrusive force generation resulted in inward flow of a matrix along the perpendicular axis. Together, these simulation results indicate that both elongation of interpolar spindles and contraction of cytokinetic ring drive mitotic elongation during cell division in collagen gels.

## 3 Discussion and Conclusion

**Figure 6:**
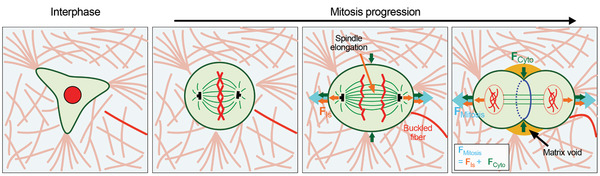
Proposed mechanisms for pushing forces that drive mitotic elongation in collagen gels. During interphase, cells are able to spread in collagen gels and remodel the surrounding network. When cells enter mitosis, they become rounded and prepare for division. From anaphase to T/C, cells generate extracellular protrusive forces through elongation of the interpolar spindle, which couples to the surrounding collagen matrix through the astral microtubules, and contraction of cytokinetic ring, which drives mitotic expansion due to volume conservation. Such protrusive forces push away the surrounding collagen fibers along the mitotic axis, and thereby generate space for mitotic elongation to proceed.

Taken together, our studies reveal that cells undergoing mitosis in collagen gels, a stroma mimicking ligand‐rich microenvironment, generate pushing forces that clear space in the surrounding matrix for mitotic elongation. Deformation of collagen networks by pushing forces increased along the mitotic axis as cells proceeded through mitosis, with the greatest deformation occurring at AnaB and T/C. Mechanistically, extracellular pushing force generation during single cell division was found to arise from interpolar spindle elongation, which could couple to the surrounding matrix through astral microtubules, and lateral contraction by cytokinetic ring, which expanded the mitotic axis due to volume conservation (**Figure** **6**), similar to mitotic force generation in alginate gels.^[^ ^8^ ^]^ We note the possibility that the two mechanisms could be interdependent. In addition, other mechanisms, such as mechanochemical signaling pathways and positive feedback mechanisms, could also be involved in pushing force generation during mitosis. Matrix deformation was found to occur at similar levels in collagen gels of various densities. Since gels of collagen with higher density exhibit higher stiffness, such similar matrix deformations indicate that mitotic cells generate increased mitotic forces in denser collagen gels. Finally, levels of matrix deformation generated during mitotic elongation were similar when cells were allowed to spread prior to mitosis and when matrix degradation was inhibited. The role of cell spreading and matrix degradation in matrix remodeling has been suggested in many different contexts, for example, invadopodia formation,^[^ ^22^ ^]^ and creation of space for division.^[^ ^17^ ^]^ However, our results indicate that neither cell spreading nor matrix degradation is required for force generation during mitotic elongation. It is possible, and perhaps likely, that there are many subtle differences underlying cell–matrix interactions during mitosis when cells are allowed to spread and remodel the matrix prior to mitosis, versus when they undergo mitosis from the rounded state. In addition, variation in collagen architecture, including pore size, collagen fiber diameter and length, and the degree of intermolecular and intramolecular crosslinking, due to changes in collagen density could have some impact on mitosis and the process of mitotic elongation.

Our finding of extracellular pushing forces driving mitotic elongation is expected to be relevant to cell division in tissues, but would not have been observed in 2D culture studies. Most studies on cell division have been conducted in sparse 2D culture, where cells simply release from the substrate and undergo mitotic elongation without encountering any resistance, and thus not require force. However, in many tissue contexts, such as a tumor growing within a collagen‐rich stromal matrix, cells are three‐dimensionally surrounded by their extracellular matrix. In such cases, the surrounding matrix would be expected to provide a mechanical barrier to mitotic elongation. The gels of collagen studied used for our studies provide a confining microenvironment to mitotic cells along with physiologically relevant signaling cues. While most of this study was performed using MDA‐MB‐231 cells, force generation during mitosis was also demonstrated for MCF10A cells, nontumorigenic breast cells, revealing that the finding is not specific to only cancer cells.

Our finding of the crucial role of extracellular force generation in division, independent of cell spreading, complements two recent studies highlighting the connection between cell spreading and mitosis. In a study of 3T3 fibroblasts cultured in fibrin gels, it was found that the cells were able to divide while maintaining long and thin protrusions along the direction of spreading.^[^ ^17^ ^]^ The dividing fibroblast cells exerted contractile forces at the tip of protrusions, and these forces guided the orientation of the cell division axis. Another study examining division of HT1080 fibrosarcoma cells in collagen gels revealed that some spread cells remained spread while proceeding through mitosis, with the division axis directed by cell spreading.^[^ ^18^ ^]^ While these studies show that cell spreading can contribute to mitotic elongation in some contexts, our findings suggest that this contribution may not be required. Our finding that cells are able to create space for mitotic elongation during mitosis via pushing force generation suggest that the division axis does not need to be predetermined by cell spreading or matrix remodeling before mitosis. This idea is consistent with other studies reporting that the division axis reorients in response to external forces.^[^ ^34^, ^35^, ^36^ ^]^ Thus, extracellular force generation during mitotic elongation may be an essential mechanism that enables plasticity of mitotic axis orientation.

The observation of clear matrix voids along the perpendicular axis of dividing cells implicates that protrusive extracellular force generation may drive the inward movement of the surrounding matrices. A recent study examining division of zebrafish epicardial cells in monolayer found that their cytokinetic ring is directly anchored to the underlying substrate and contraction of cytokinetic ring generates inward traction forces to the interface between two daughter cells.^[^ ^27^ ^]^ While we also observed inward movement of matrix along the perpendicular axis of dividing cells, the clear matrix voids between dividing cells and the surrounding collagen networks indicate that cytokinetic ring of dividing cells in collagen gels is not directly coupled to the surrounding fibers, and therefore the contraction of cytokinetic ring does not account for the observed inward movement of matrix. Indeed, our simulations show that when elongation of cell body along the mitotic axis increases, the magnitude of inward flow along the perpendicular axis increases, suggesting the role of mitotic elongation of dividing cells in generating the inward flow in the perpendicular axis (Figure 5H; Figure S13D,E, Supporting Information). This observation highlights the long‐range mechanical connectivity of collagen networks, as has been remarked on previously.^[^ ^37^ ^]^

In addition, our results provide fundamental new insights into how tumor cells are able to proliferate and eventually form multicellular tumors in dense collagen‐rich tissues. It has been reported that various types of solid tumors are under mechanical compression.^[^ ^5^ ^]^ Therefore, tumors must generate outward forces to overcome the mechanical constraint imposed by the surrounding tissues and to create space for sustained division of tumor cells. While it is known that cells are able to generate actomyosin mediated contractile forces through integrins, such contractile forces cannot account for outward force generation. Single‐cell studies suggest multiple possible sources of outward force generation during tumor growth including forces generated during cell growth in the G1 phase of the cell cycle,^[^ ^6^ ^]^ forces of mitotic swelling,^[^ ^38^, ^39^, ^40^ ^]^ and, as studied here, forces generated during mitotic elongation. However, it should be noted that in multicellular tumors, the origins of mechanical force for expansion could be more complex than the division of single cells, due to the presence of other cells surrounding the dividing cells and consideration of cell–cell interactions and coordinated cell movements. Broadly, this work reveals the biophysical mechanisms of cell division in collagen gels and highlights the complexity of cell division occurring in physiological microenvironments.

## 4 Experimental Section

##### Cell Culture

MDA‐MB‐231 human cells transfected with GFP‐labeled *α*‐tubulin and RFP‐labeled histone were kind gifts from B. Weaver (University of Wisconsin‐Madison). The cells were cultured in high glucose Dulbecco's modified Eagle's medium (DMEM) containing 10% fetal bovine serum (Hyclone) and 1% penicillin/streptomycin (Gibco/Thermo Fisher Scientific). MCF10A cells were cultured in DMEM/F12 50/50 medium supplemented with 5% horse serum, 20 ng mL^−1^ EGF, 0.5 µg mL^−1^ hydrocortisone, 100 ng mL^−1^ cholera toxin, 10 µg mL^−1^ insulin, and 100 U mL^−1^ Pen/Strep.

##### Cell Experiments

For cell division assays on cells that were not spread, MDA‐MB‐231 cells were treated with thymidine at 2 × 10^−3^ m for one day to arrest at G1/S phase, released and then treated with STLC at 20 × 10^−6^ m for one day to arrest at prometaphase. The cells arrested at prometaphase were then released from STLC and suspended in complete medium. Next, type I collagen solution was prepared by diluting with 10 × DMEM, 1 × DMEM, and the cell suspension. The final concentration of collagen gels was 1, 3, and 5 mg mL^−1^, and the final cell density was 0.1–0.5 million cells mL^−1^. The pH of the solution was adjusted to 8.5 by adding NaOH. The collagen solution containing cells was then deposited on a cell‐culture plate equipped with cover glass at the bottom, and allowed to be polymerized at 37 °C for 45–60 min. To perturb cytokinetic ring contraction, blebbistatin (100 × 10^−6^ m) was added to the collagen solution. Once collagen solution was fully gelled, live‐cell imaging was performed. As an alternative to thymidine/STLC for cell cycle synchronization, the cells were treated with Cdk1 inhibitor RO‐3306 (9 × 10^−6^ m) for 18 h.^[^ ^41^ ^]^

For cell division assays on cells that were spread, cells were treated with thymidine at 2 × 10^−3^ m for one day, released, and encapsulated into collagen gels of varying density. When collagen solution containing cells became fully polymerized, complete medium containing STLC was added to the gels and cells were incubated for one day. Next day, the medium was replaced with standard to release cells from STLC, and live‐cell imaging was performed. For inhibition of MMPs, a cocktail of protease inhibitors, including marimastat (20 × 10^−6^ m), E‐64 (20 × 10^−6^ m), aprotinin (0.7 × 10^−6^ m), and leupeptin (2 × 10^−6^ m), was added to the medium along with STLC. This combination of inhibitors was previously shown to potently inhibit MMP activity.^[^ ^28^ ^]^ To partially inhibit kinesin‐5 activity during mitosis, STLC (10 × 10^−6^ m) was added to the medium.

##### Live‐Cell Imaging

Cells undergoing mitosis were imaged using confocal microscopy (Leica, SP8) under normal cell culture conditions (37 °C, 5% CO_2_). Cells at prometaphase were first located through the eyepiece. Once cells proceeded mitosis, they were imaged using time‐lapse microscopy with a 25X/0.95NA water‐immersion objective at excitation wavelengths of 488 nm for GFP‐labeled *α*‐tubulin, 555 nm for RFP‐labeled histone, and 639 nm for confocal reflectance of collagen fibers.

For live‐cell imaging from interphase, cells were treated with thymidine (2 × 10^−3^ m) for one day, released, and encapsulated in collagen gels of 3 mg mL^−1^. Live‐cell imaging was then performed after 10 h of culturing in the presence of RO‐3306 (9 × 10^−6^ m). Cells with spreading morphology were selected for imaging. After 5 h of imaging, cells were released from RO3306 and allowed to proceed through mitosis.

For cell division assay with MCF10A cells, MCF10A cells labeled with RFP‐histone were treated with RO‐3306 (9 × 10^−6^ m) and with SiR‐tubulin (100 × 10^−9^ m) for 18 h. Cells were then released and encapsulated into collagen gels of 3 mg mL^−1^. When the collagen solution containing cells became fully polymerized, complete medium was added to the gel and cells and live‐cell imaging was performed.

##### Mechanical Characterization

For mechanical characterization of collagen gels, rheological testing was performed using an AR‐G2 stress‐controlled rheometer (TA Instruments, Newcastle, DE) equipped with 25 mm diameter top and bottom plate geometry. Poly‐l‐lysine‐coated coverslips (25 mm; Neuvitro, El Monte, CA) were attached to the surface of the rheometer geometry with double‐sided tape to enhance the attachment of collagen gels to the surface. Type I Collagen (Corning, Corning, NY) was prepared in ice by diluting with 10× and 1× DMEM to achieve a final concentration of 1, 3, and 5 mg mL^−1^. The pH of collagen presolution was adjusted to 8.5 by adding NaOH. Note that the mechanical properties of collagen gels formed with a pH of 7.5 exhibited similar to those with pH of 8.5 (Figure S1, Supporting Information). The collagen solution was immediately deposited between the rheometer plates, and mineral oil (Sigma Aldrich) was then deposited around the exposed surface of the solution to prevent dehydration. The collagen solution was allowed to gel for 1 h at 37 °C. During gelation, the storage modulus was monitored by applying oscillations at a strain of 0.01 and frequency of 1 rad s^−1^. The final value of storage modulus during gelation was used for reporting collagen gel modulus.

Once the collagen solutions became fully gelled, creep and recovery tests were performed to assess plasticity. In creep and recovery tests, a constant stress was applied for 300 s while strain in response to the stress was measured over time. The value of stress was adjusted so that the resultant strain at the end of creep tests was ≈30%. Next, the stress was removed in recovery tests, and the strain was monitored for 1 h. The degree of plasticity was defined as the ratio of the remaining strain after recovery tests to the maximum strain at the end of the creep tests, or

(1) $$\mathrm{Degree}\;\mathrm{of}\;\mathrm{plasticity}=\frac{\varepsilon_{\mathrm{remaining}}}{\varepsilon_{\mathrm{maximum}}}$$

##### Laser Ablation Experiments

Laser ablation experiments were performed using a multiphoton laser scanning confocal microscope (Zeiss, LSM 780) under normal cell culture conditions (37 °C, 5% CO_2_) at the Stanford Cell Sciences Imaging Facility. For this experiment, fluorescent microbeads (Thermo Fisher Scientific) were embedded in collagen gels to clearly visualize matrix deformation, and 3 mg mL^−1^ collagen gels were used, in order to firmly embed microbeads in the network. The size of microbeads was measured to be 1.5 µm in average (Figure S8A,B, Supporting Information), and this was similar to the pore size of ≈3 mg mL^−1^ collagen gels.^[^ ^30^, ^31^ ^]^ Therefore, microbeads possessing a size larger than the pore size of the collagen gels were expected to be physically trapped in the collagen network. A kymograph of microbeads further confirmed that microbeads remained in their original position throughout the experiments (Figure S8C,D, Supporting Information). After confirming that microbeads were embedded in the collagen gels, cell experiments were performed. Live‐cell imaging of mitotic cells was conducted through a 20×/0.8NA water‐immersion objective at excitation wavelengths of 405 nm for microbeads, 488 nm for GFP‐labeled *α*‐tubulin and 555 nm for RFP‐labeled histone. Once cells entered anaphase, laser ablation was performed to sever mitotic spindles using a Mai Tai DeepSee (Spectra‐Physics) laser at a wavelength of 800 nm with 90–100% of maximum power of 2.4 W.

##### Mitotic Stage Determination

The stage of mitosis was determined manually following the previous criteria: metaphase—chromosomes were lined up along the metaphase plate; anaphase A—chromosomes were initiated to segregate; anaphase B—cell body and interpolar spindles were elongated and cleavage furrow was initiated; telophase/cytokinesis—the cleavage furrow ingression was fully developed and mid body was formed.

##### Quantification of Reflectance Intensity

Reflectance intensity of collagen fibers was quantified using ImageJ. Regions of interest with a 16 µm diameter for the evaluation of reflectance intensity were randomly selected at regions near cells and at regions far from cells. The reflectance intensity for each region of interest was obtained and the mean value was reported.

##### Image Analysis

The length of cells along the mitotic axis, the length of spindle, and the width perpendicular to the mitotic axis at each stage of division were measured using ImageJ. The size of fluorescent microbeads was measured using particle analysis in ImageJ.

##### Matrix Deformation Calculation

Matrix deformation of collagen gels was acquired through image analyses using Image J and MATLAB. First, images of mitotic cells were collected and registered using Image J plugin (StackReg) to correct for drift. Next, the registered images were used to calculate matrix deformation by tracking collagen fibers using particle image velocimetry algorithm (PIVlab; open source code for MATLAB). Erratic displacements, including displacements of beads inside the cell body, were replaced by the averaged values of surrounding displacements. Matrix deformation fields were created with one pass of smoothing to improve consistency of displacements. Maximum matrix deformation was selected from within ≈15 µm around the cells. It was noted that estimation of forces from collagen deformation was a challenging problem due to the nonlinear elasticity, viscoelasticity, plasticity, and heterogeneity of collagen gels, as described in previous studies.^[^ ^42^, ^43^ ^]^ Therefore, in this study, characterizing matrix deformation without further analysis or processing was focused on, since matrix deformation only occurred as the result of cellular forces.

##### Volume Measurement

For cells undergoing mitosis, 3D imaging was conducted using confocal microscopy (Leica, SP8) with 2 µm intervals in the *z*‐axis between images. The images collected from *z* stack confocal microscopy were three‐dimensionally reconstructed using IMARIS (Bitplane). The total volume of cells at different mitotic stages was also calculated in IMARIS, and normalized by the volumes at T/C.

##### Kymograph Analysis

Kymographs of fluorescent microbeads were created using ImageJ for cells subjected to laser ablation. Lines were drawn on top of microbeads of interest in a stack of images. Pixels that the lines passed through were reorganized to generate a kymograph using plug‐in of ImageJ.

##### Computational Model—Brownian Dynamics with the Langevin Equation

A matrix was created consisting of crosslinked fibers with or without a cell via an agent‐based computational model based on Brownian dynamics. The values of all parameters are listed in Table S1 in the Supporting Information. In the simulation model, matrix fibers consisted of cylindrical elements that were serially connected to each other. Crosslinkers were comprised of two cylindrical elements. The cell membrane was coarse‐grained into serially connected rectangular solid elements. Nodes connecting two membrane elements were defined by only *x* and *y* positions, whereas nodes constituting fibers and crosslinkers had *x*, *y*, and *z* positions. Displacements of all nodes were determined by the Langevin equation with inertia neglected

(2) $$F_{i}-\xi_{i}\frac{dr_{i}}{dt}+F_{i}^{T}=0$$

where **r** *_i_* is a position vector of the *i*th node, *ζ_i_* is a drag coefficient, *t* is time, **F** *_i_* is a deterministic force, and **F** *_i_* ^T^ is a stochastic force satisfying the fluctuation–dissipation theorem^[^ ^44^ ^]^

(3) $$〈F_{i}^{T}\left(t\right)F_{j}^{T}\left(t\right)〉=\frac{2k_{B}T\xi_{i}\delta_{\mathrm{ij}}}{\Delta t}\delta$$

where *δ_ij_* is the Kronecker delta, ***δ*** is a second‐order tensor, and Δ*t* is a time step. Drag coefficients of cylindrical elements were calculated using an approximated form for a cylindrical object^[^ ^45^ ^]^

(4) $$\xi_{i}=3\pi \mu r_{c,i}\frac{3+2r_{0,i}/r_{c,i}}{5}$$

where *μ* is the viscosity of medium, and *r*_0,_ *_i_* and *r*_c,_ *_i_* are length and diameter of cylindrical elements, respectively. For simplicity, the drag coefficient of membrane elements was the same as that of fiber elements. Positions of all nodes were updated via Euler integration scheme

(5) $$r_{i}\left(t+\Delta t\right)=r_{i}\left(t\right)+\frac{dr_{i}}{dt}\Delta t=r_{i}\left(t\right)+\frac{1}{\xi_{i}}\left(F_{i}+F_{i}^{T}\right)\Delta t$$

Deterministic forces included extensional and bending forces maintaining equilibrium lengths and angles as well as repulsive forces representing volume‐exclusion effects between membrane elements and the elements that represented fibers and crosslinkers. The extensional and bending forces for fibers and crosslinkers originated from harmonic potentials

(6) $$U_{s}=\frac{1}{2}\kappa_{s}{\left({r-r}_{0}\right)}^{2}$$

(7) $$U_{b}=\frac{1}{2}\kappa_{b}{\left(\theta -\theta_{0}\right)}^{2}$$

where *κ*_s_ and *κ*_b_ are extensional and bending stiffnesses, *r* and *r*_0_ is instantaneous and equilibrium lengths of cylindrical elements, and *θ* and *θ*_0_ are instantaneous and equilibrium angles defined by adjacent elements. An equilibrium length of fiber elements (*r*_0,f_ = 1 µm) and an equilibrium angle defined by two adjacent fiber elements (*θ*_0,f_ = 0 rad) were maintained by extensional (*κ*_s,f_) and bending stiffnesses (*κ*_b,f_) of fibers, respectively. The reference value of *κ*_b,f_ corresponds to the persistence length of ≈100 µm. An equilibrium length of crosslinker arm (*r*_0,xl_ = 200 nm) was maintained by extensional stiffness (*κ*_s,xl_), and an equilibrium angle between two arms of each crosslinker (*θ*_0,xl,1_ = 0 rad) and an equilibrium angle between a crosslinker arm and the axis of a fiber where the arm was bound (*θ*_0,xl,2_ = *π*/2 rad) were regulated by two bending stiffnesses (*κ*_b,xl,1_ and *κ*_b,xl,2_). Forces exerted on fiber elements by bound crosslinkers were distributed onto two nodes located at the ends of fiber elements.

An equilibrium angle between adjacent membrane elements (*θ*_0,m_ = 0 rad) was maintained by bending stiffness (*κ*_b,m_), and an equilibrium length of membrane elements (*r*_0,m_) was maintained by extensional stiffness (*κ*_s,m_). Volume encapsulated by the membrane was conserved as explained in detail later. Repulsive forces between membrane elements and the elements representing matrix fibers and crosslinkers led to deformation of the surrounding matrix while the membrane changed its shape. A minimum distance between a membrane element and a cylindrical segment accounting for fibers or crosslinkers, *r*_12_, was computed. Then, repulsive forces were determined by a harmonic potential

(8) $$U_{r}=\left\{\begin{array}{cc} \frac{1}{2}k_{r}{\left(r_{12}-r_{c}\right)}^{2} & \mathrm{if}\;r_{12}<r_{c}\\ 0 & \mathrm{if}\;r_{12}\geq r_{c} \end{array}\right.$$

where *κ*_r_ is strength of repulsive force, and *r*_c_ is a critical distance.

##### Computational Model—Stress Relaxation Test

To evaluate the rheological properties of the matrix, a matrix without a cell was created (Figure S12, Supporting Information). Fiber formation began from emergence of one fiber element whose length was 1 µm. The element was elongated by adding identical cylindrical elements. After all fibers were formed, the average length of fibers was 9.3–11.6 µm. Crosslinkers bound to binding sites located every 100 nm on matrix fibers and also unbound from fibers in a force‐dependent fashion following Bell's law^[^ ^46^ ^]^

(9) $$k_{u}=\left\{\begin{array}{cc} k_{0,u}\exp\left(\frac{\lambda_{u}\left|{\vec{F}}_{s,x1}\right|}{k_{B}T}\right) & \mathrm{if}\;r\geq r_{0,X1}\\ k_{0,u} & \mathrm{if}\;r<r_{0,X1} \end{array}\right.$$

where ${\vec{F}}_{s,X1}$ is a vector representing a spring force acting on a crosslinker arm, *k*_0,u_ is a zero‐force unbinding rate, *λ*_u_ is sensitivity to the spring force, and *k*_B_ *T* is thermal energy. Only when the spring force is tensile, an unbinding rate, *k*_u_, is enhanced beyond its base rate, *k*_0,u_.

First, a matrix within a 3D rectangular domain (50 × 50 × 1 µm) with a periodic boundary condition in *x* and *y* directions was preassembled, via fiber formation and binding of crosslinkers to fibers. Then, the preassembled matrix was loaded at the beginning of simulations for stress relaxation tests. All elements that crossed the boundaries of the computational domain located in the *y* direction were removed, and nodes that constituted the removed elements were clamped to the boundaries. The top boundary was translated in the +*x* direction to reach goal shear strain level with a strain rate of 0.1 s^−1^, whereas the bottom boundary was stationary. After the shear strain reached the goal level, it was maintained at the goal level till the end of simulations. Stress acting on the top boundary was calculated by dividing the sum of all forces exerted on nodes clamped to the top boundary by the boundary area.

To compare with experimental results, fiber concentration was calculated in mg mL^−1^ as follows. First, it was assumed that fibers in the model represented collagen fibrils whose Young's modulus, *E* ≈ 30 MPa.^[^ ^47^ ^]^ To satisfy the following relationship,^[^ ^3^ ^]^ the radius of fibers (*r*_c,f_) needs to be 6.5 nm

(10) $$k_{s,f}=\frac{\mathrm{EA}}{r_{0,f}}=\frac{\pi Er_{c,f}^{2}}{r_{0,f}}$$

where *A* is the cross‐section area of fibers. Then, using the following equation^[^ ^48^ ^]^ fiber concentration (*C*) in mg mL^−1^ can be calculated

(11) $$C=\frac{\pi r_{c,f}^{2}L_{f,\mathrm{tot}}}{Vv}$$

where *L*_f,tot_ is the total fiber length in the matrix, *V* is the total volume of the matrix, and *ν* is the specific volume of collagen, 0.73 mL g^−1^.

##### Computational Model—Cytokinesis

To investigate cytokinesis occurring in a collagen matrix, a cell membrane surrounded by a matrix within a cylindrical computational domain was simulated whose radius and height were 50 and 1 µm, respectively. At the beginning of each simulation, a circular membrane whose radius was 10 µm was positioned at the center of the domain. The number and equilibrium length of membrane elements were 158 and 400 nm, respectively. As a result, the interconnected rectangular solid elements created a wall dividing a domain space into intracellular and extracellular spaces. Then, a matrix was formed around the membrane as explained above.

To mimic forces exerted by cytokinetic ring, each of the membrane nodes located within 0.5 µm from the equator of the membrane was subjected to a constant inward force in the *y* direction, *F*_Cyto_ (Figure 5A). The total amount of forces acting on the membrane nodes located on each side was 100 pN. When the membrane nodes approached the mitotic axis of the membrane within 1 µm, the forces were not applied to the nodes. For simulating forces from mitotic spindles, membrane nodes within 5 µm from the mitotic axis of the membrane underwent constant outward forces in the *x* direction, *F*_Is_ (Figure 5F). The total amount of forces acting on membrane nodes on either side was 100 pN. It was assumed that cell volume was conserved during cytokinesis. The model approximately simulated the cross‐section of a cell undergoing cytokinesis. It was observed that a cell shape in the model remained nearly symmetric along its long axis during cytokinesis. Thus, at each time point, the volume of a 3D cell reconstructed by rotating the 2D cell shape about the long axis was calculated. If the instantaneous cell volume was not equal to initial volume, transverse forces were applied to all membrane segments inward or outward to restore the initial volume. Repulsive forces acting between membrane elements and matrix elements led to deformation of the surrounding matrix when the membrane changed its shape due to forces from the ring and the mitotic spindle.

##### Statistical Analysis

Data are presented as mean ± SD as specified in the figure legends and analyzed with Graph Pad Prism 8. The number of cells analyzed and preprocessing normalization of data are indicated in the corresponding figure legends. For statistical analysis, Student's *t*‐tests were used to compare two groups using GraphPad. One‐way analysis of variance with Tukey's multiple comparison was used to compare more than two groups. n.s. not significant, ^*^ *p* < 0.05, ^**^ *p* < 0.01, ^***^ *p* < 0.001, and ^****^ *p* < 0.0001.

## Conflict of Interest

The authors declare no conflict of interest.

## Author Contributions

S.N. and Y.‐H.L. contributed equally to this work. S.N., Y‐H. L., and O.C. designed the experiments and analyzed the data. S.N. and Y.‐H.L. performed experiments. T.K. ran simulations. S.N., Y.‐H. L., T.K., and O.C. wrote the manuscript.

## Supporting information

Supporting Information

Supplemental Movie 1

Supplemental Movie 2

Supplemental Movie 3

Supplemental Movie 4

Supplemental Movie 5

Supplemental Movie 6

Supplemental Movie 7

Supplemental Movie 8

Supplemental Movie 9
