# Supplementary material for: Cellular Pushing Forces during Mitosis Drive Mitotic Elongation in Collagen Gels
Source: Adv Sci (Weinh). 2021 Jan 4;8(4):2000403. doi: 10.1002/advs.202000403 (PMC7887597; doi:10.1002/advs.202000403)
Supplement: Supplementary file 1 — Supporting Information [file ADVS-8-2000403-s001.pdf]

© 2020 Wiley-VCH GmbH

ADVANCED SCIENCE
Open Access

Supporting Information

for *Adv. Sci.*, DOI: 10.1002/advs.202000403

Cellular pushing forces during mitosis drive mitotic elongation in collagen gels

Sungmin Nam, Yung-Hao Lin, Taeyoon Kim, Ovijit Chaudhuri**

Supplementary Materials

Cellular pushing forces during mitosis drive mitotic elongation in collagen gels.

Sungmin Nam[1,2,3,†], Yung-Hao Lin[4,†], Taeyoon Kim[5*], Ovijit Chaudhuri[1*]

[1]Department of Mechanical Engineering, Stanford University, CA, USA.

[2]Harvard John A. Paulson School of Engineering and Applied Sciences, Harvard University, Cambridge, MA, USA.

[3]Wyss Institute for Biologically Inspired Engineering, Cambridge, MA, USA.

[4]Department of Chemical Engineering, Stanford University, CA, USA.

[5]Weldon School of Biomedical Engineering, Purdue University, IN, USA.

[†]These authors contributed equally: Sungmin Nam and Yung-Hao Lin
[*]Address correspondence to Taeyoon Kim and Ovijit Chaudhuri

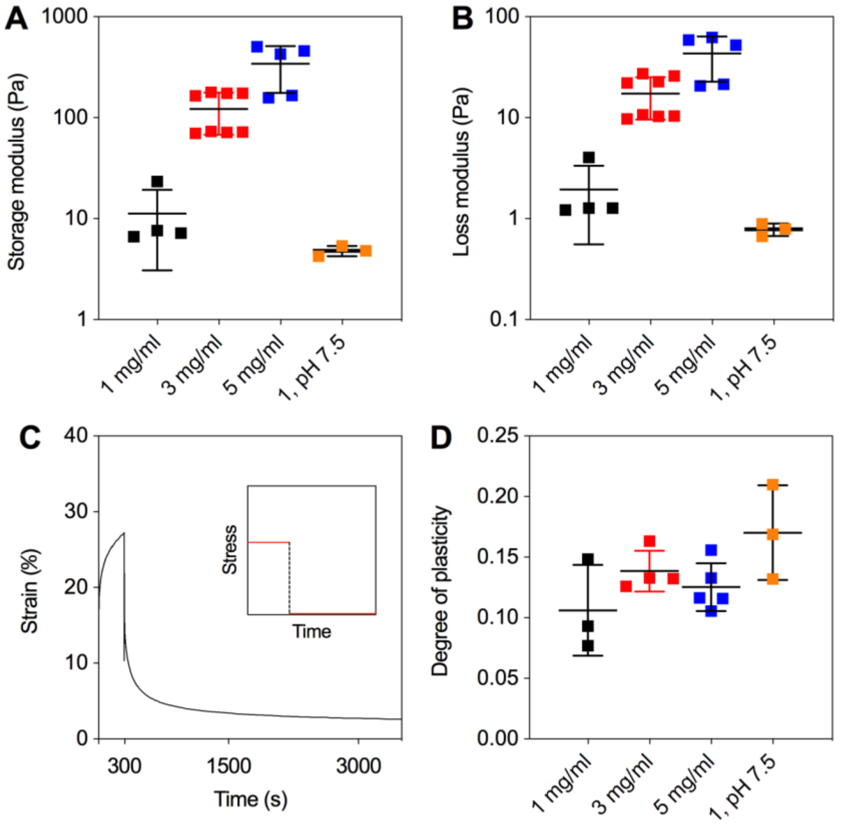

Fig. S1. Mechanical characterization of collagen gels. **A**, Storage and **B**, loss modulus of collagen gels with varying density and pH. **C**, A representative result from creep and recovery tests. Inset, a profile of stress over time in creep and recovery tests. A constant stress is applied to a sample during creep tests, and then removed after recovery tests. **D**, Degree of plasticity of collagen gels with varying density and pH. Data are presented as mean ± SD.

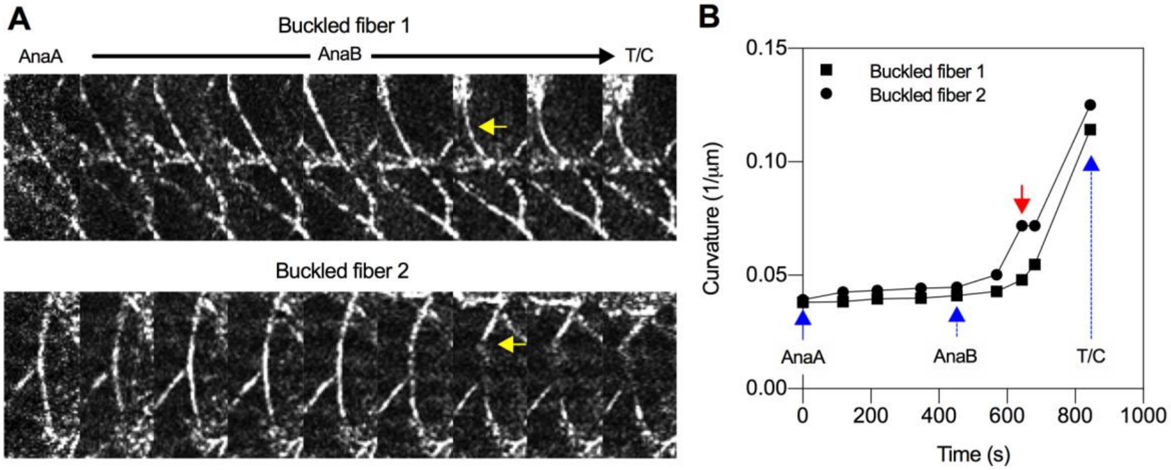

Fig. S2. Buckling of collagen fibers occurs during mitosis. A, Buckling of collagen fibers by a cell undergoing mitosis in the low-density collagen gels. The region of the buckled collagen fibers is indicated in Figure 1G. The yellow arrows represent the onset of buckling. **B**, Quantification of curvature of collagen fibers as a function of time. The red arrow indicates the onset of buckling. See Video 2.

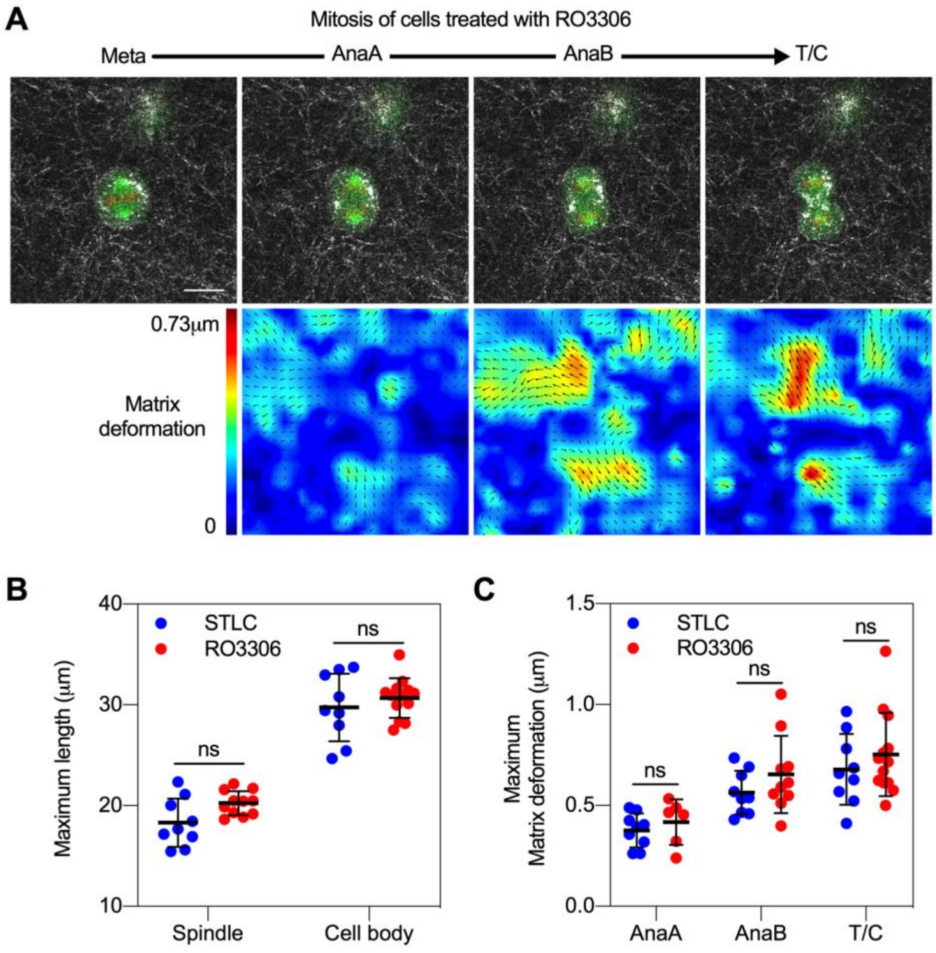

Fig. S3. Method for synchronization does not alter force generation during mitosis in collagen gels. A, Fluorescence images of a dividing cell synchronized with RO3306 along with reflectance images of collagen fibers (top row) and corresponding matrix displacement maps (bottom row). Scale bars, 10 μm. **B**, Comparisons of maximum spindle length and cell body between cells synchronized with STLC and RO3306 (n = 9 – 13, N > 3). **C**, Comparisons of maximum matrix deformation at each mitotic stage (n = 9 – 13, N > 3). Data are presented mean ± SD. Student's t-tests were used; n.s. not significant.

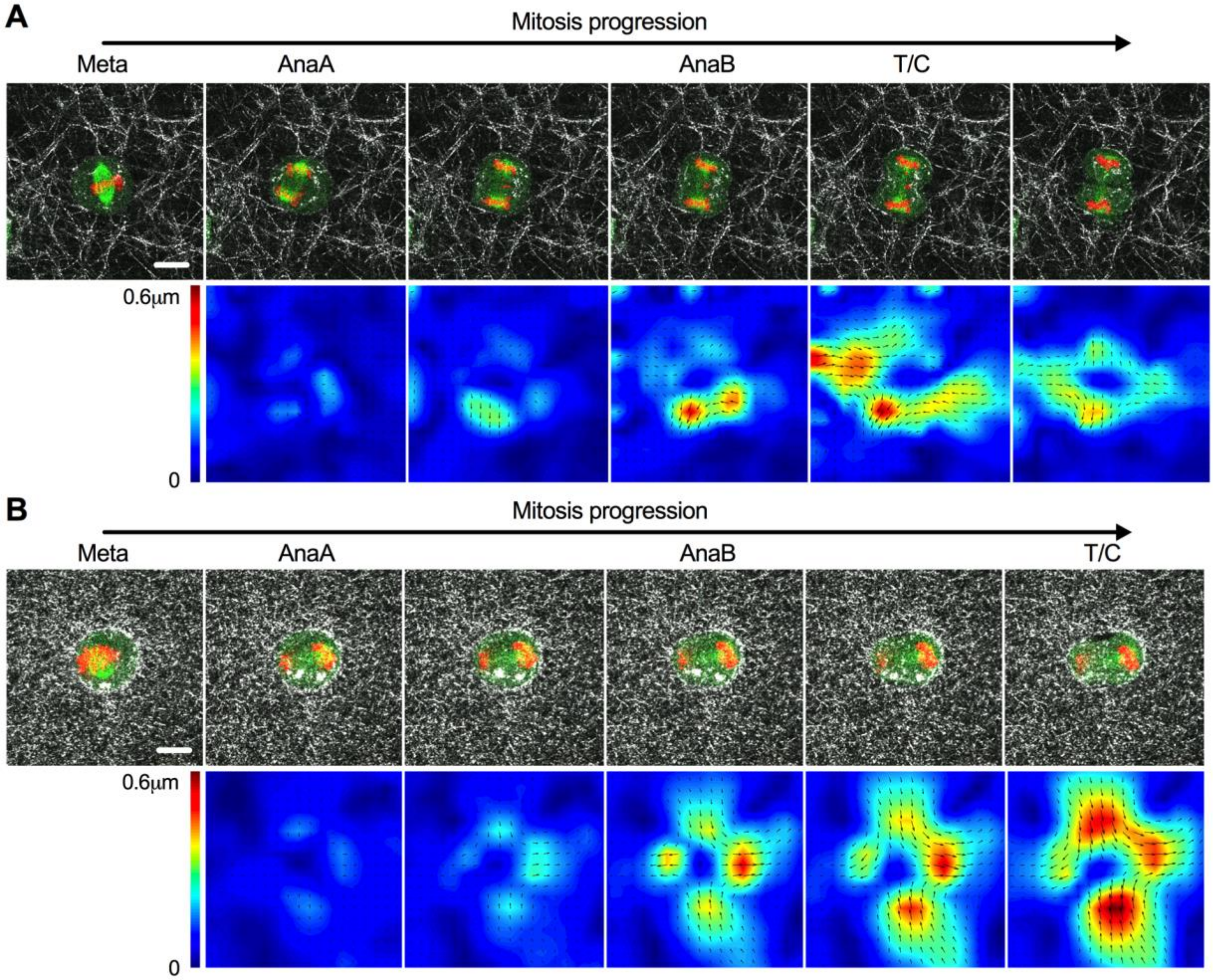

Fig. S4. Cell division in collagen gels with varying density. Cells dividing in collagen gels of **A**, 3 mg/ml and **B**, 5 mg/ml. Fluorescence images of a dividing cell along with reflectance images of collagen fibers (top row) and corresponding matrix displacement maps (bottom row). See Video 3 and 4. Scale bars, 10 μm.

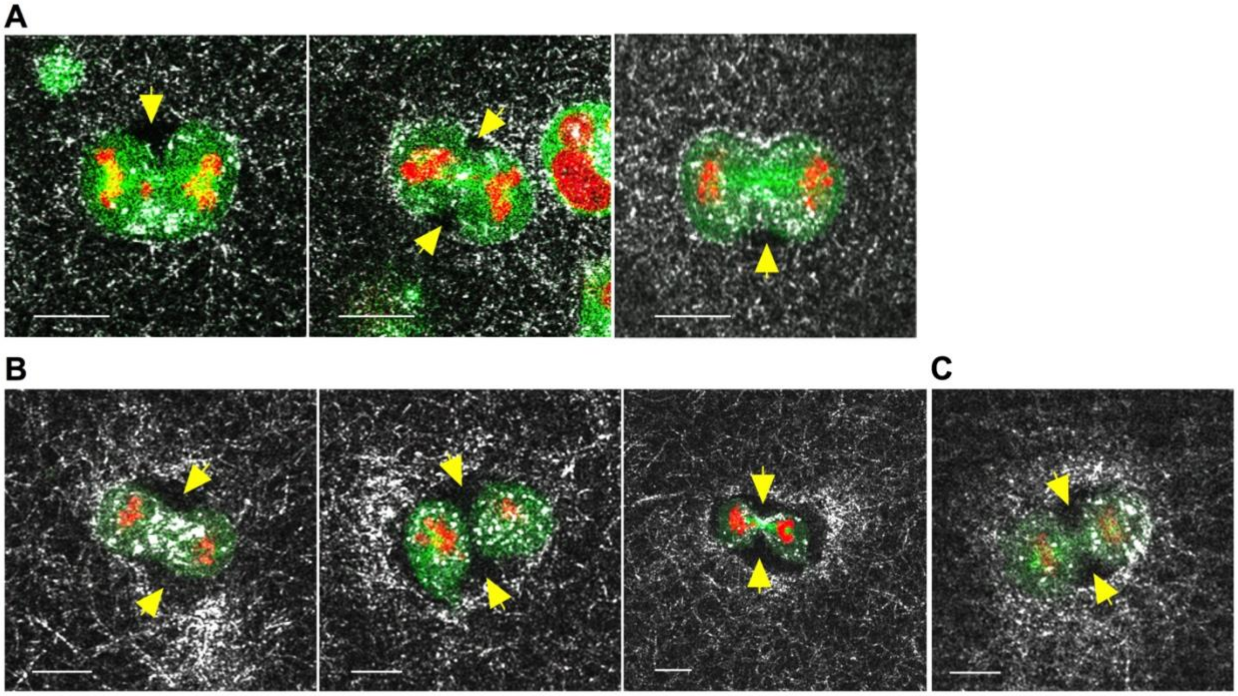

Fig. S5. Matrix voids observed adjacent to cytokinetic ring of mitotic cells. A, A gallery of cells dividing in 5 mg/ml collagen gels. **B,** A gallery of cells that were cultured in collagen gels of 1 mg/ml for one day and then divided. Yellow arrows indicate the regions of matrix voids. **C,** An image of a dividing cell that was cultured in collagen gels of 1mg/ml for one day in the presence of MMP inhibitors. Yellow arrows indicate where matrix voids were observed. Scale bars, 10 μm.

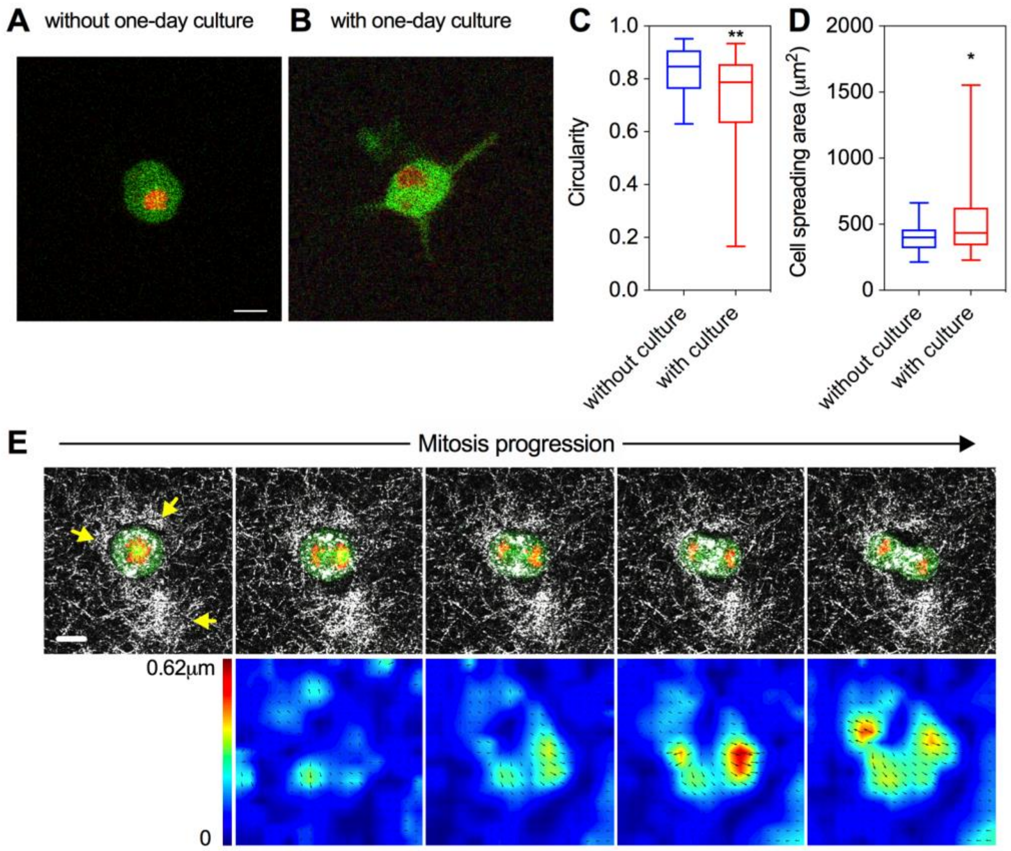

Fig. S6. Comparison of cell morphology between cells with and without one-day culture. A, Fluorescence images of an interphase cell that was imaged shortly after encapsulation in collagen gels. **B,** Fluorescence images of an interphase cell that was cultured in collagen gels for one day and then imaged. During the culture, cells were able to spread. **C,** Quantification of circularity of cells with and without one-day culture (n = 31 – 103, N > 3). The value of one indicates a perfect circle. **D,** Cell spreading area with and without one-day culture (n = 31 – 103 , N > 3). Data are presented as box and whiskers plot with min and max. **E**, Fluorescence images of a cell dividing in the low-density collagen gels along with reflectance images of collagen fibers (top row) and corresponding matrix displacement maps (bottom row). The yellow arrows indicate densified collagen fibers. See Video 5. Student t-tests; *, P < 0.05 and **, P < 0.01. Scale bars, 10 μm.

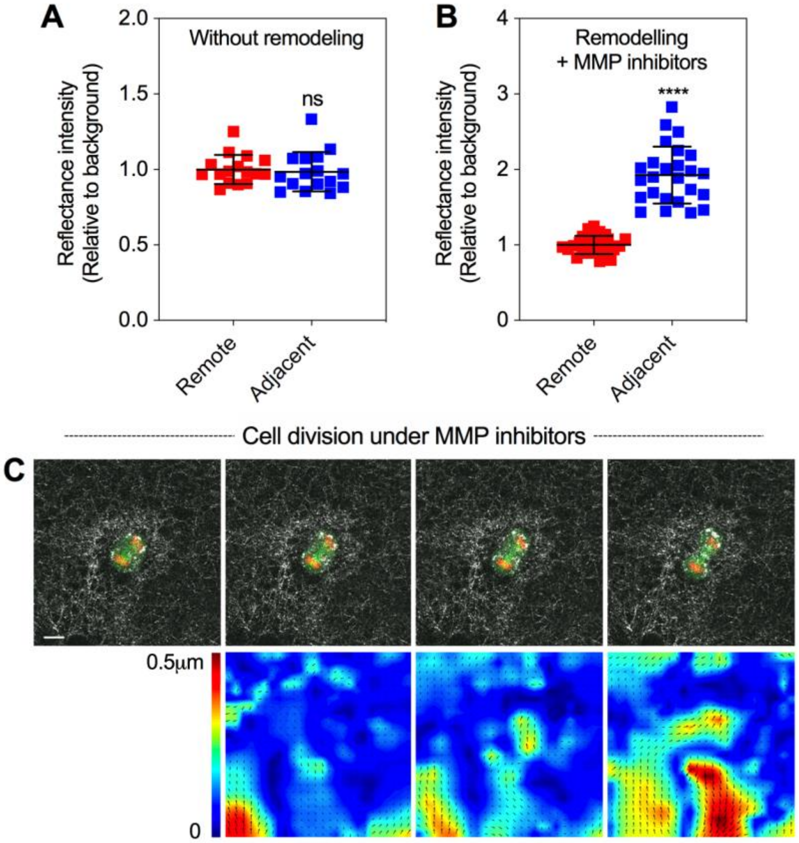

Fig. S7. Matrix deformation during mitosis are similar with and without proteolytic degradation. A, Reflectance intensity of collagen fibers for cells that divided immediately after encapsulation at regions adjacent to the cells (Adjacent, within ~20 μm) and at regions remote from the cells (Remote, greater than ~20 μm). The reflectance intensities were normalized by the average value of reflectance intensity at regions remote from the cells. **B**, Reflectance intensity of collagen fibers for cells dividing under MMP inhibitors at regions adjacent to the cells (Adjacent) and at regions remote from the cells (Remote). **C**, Fluorescence images of a dividing cell under MMP inhibitors and reflectance images of collagen fibers (top row). Matrix displacement maps corresponding to the images of the top row (bottom row). See Video 6. Scale bars, 10 μm.

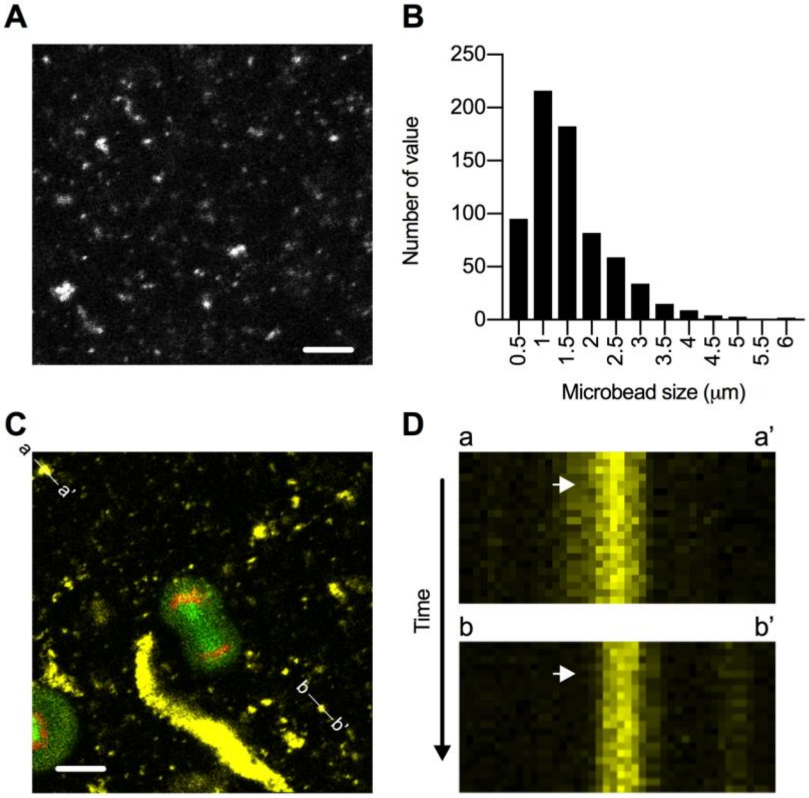

Fig. S8. Confirmation that microbeads were trapped in collagen gels. A, A fluorescent image of microbeads encapsulated in collagen gels. **B**, Histogram of microbead size. **C**, Fluorescence images of a dividing cell and microbeads. The lines tracked over time were drawn in the image. **D**, Kymographs of microbeads on the lines specified in **C**. The time scale of kymographs is ~ 2s. White arrows indicate when laser ablation was performed. Scale bars, 10 μm.

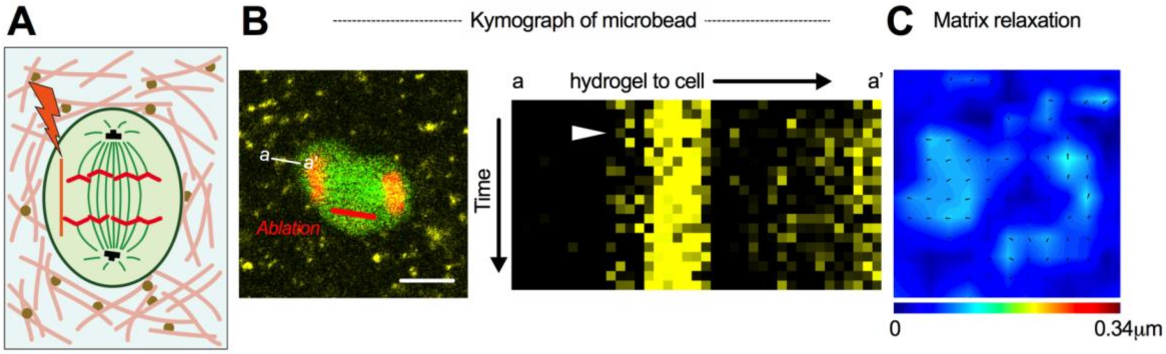

Fig. S9. Laser ablation experiments performed on region of cell body where mitotic spindle was not positioned. A, A schematic diagram of laser ablation experiments performed on where mitotic spindles was not positioned. **B**, Fluorescence images of a dividing cell and microbeads (left). The specified line tracked over time were drawn (left). A kymograph of microbeads on the specified line (right). The white arrowhead indicates when laser ablation was performed. The time scale of kymographs is ~ 2s. **C**, Matrix relaxation corresponding to the laser ablation experiment in **B**. The color bar of matrix relaxation was scaled to be the same as Figure 3D. See Video 8. A scale bar, 10 μm.

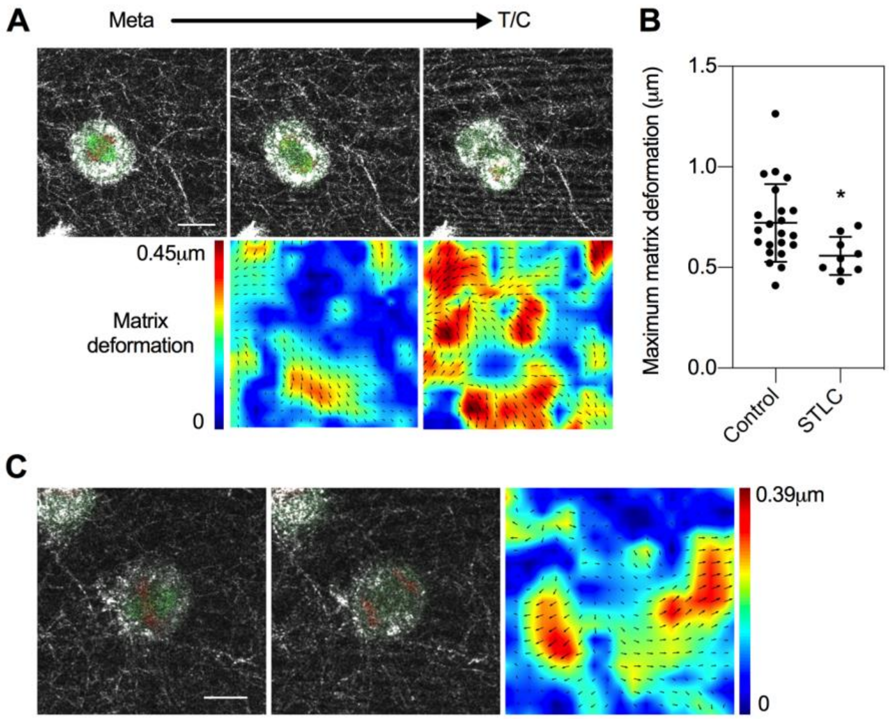

Fig. S10. Pharmacological disruption of spindle elongation and cytokinetic ring contraction decrease extracellular pushing force generation during mitosis. A, Fluorescence images of a dividing cell treated with a low concentration of STLC and reflectance images of collagen fibers (top row). Matrix displacement maps corresponding to the images of the top row (bottom row). **B**, Comparisons of maximum matrix deformation between cells treated with and without a low concentration of STLC. **C**, Fluorescence images of a dividing cell treated with blebbistatin and reflectance images of collagen fibers, and corresponding matrix displacement maps. Scale bars, 10 µm.

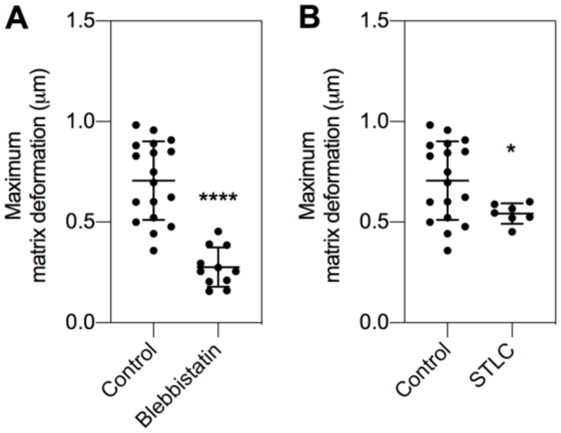

Fig. S11. Pharmacological disruption of spindle elongation and cytokinetic ring contraction also decrease pushing forces generated by cells that divide after spreading. Comparisons of maximum matrix deformation **A**, between cells treated with and without blebbistatin (n = 11 – 18, N > 3), and **B**, between cells treated with and without a low concentration of STLC (n = 7 – 18, N >3). Student t-tests; *, $P < 0.05$ and ****, $P < 0.0001$.

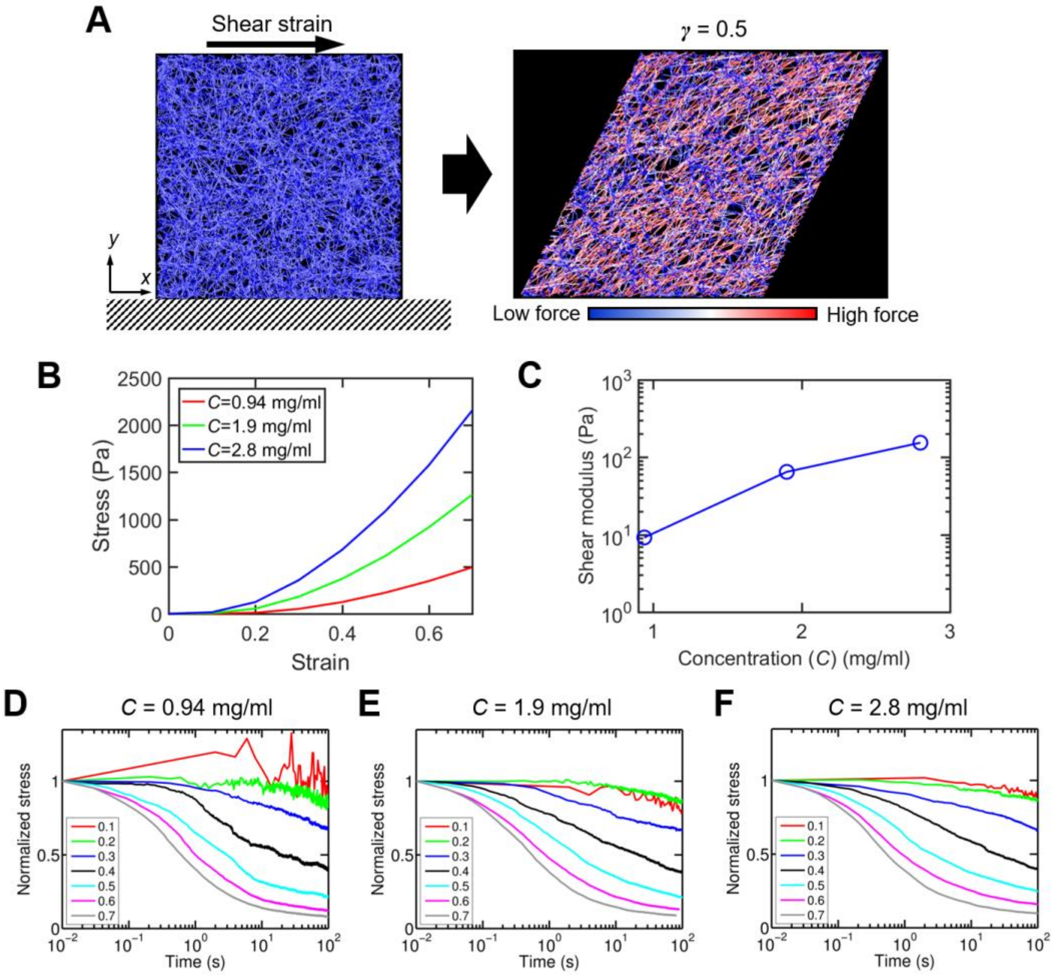

Fig. S12. Computational model of collagen gel captures the nonlinear elasticity, shear modulus, and strain-enhanced stress relaxation behavior physical collagen gels. A, Shear strain of 0.5 was applied to the top boundary of a matrix whose dimension is 50×50×1 μm^3. The bottom boundary was fixed, and a periodic boundary condition was imposed on left and right boundaries. Large shear strain resulted in tensed fibers (red) and buckled fibers (blue). **B**, Stress-strain relationships with three different fiber concentrations. All cases showed strain-stiffening as has been observed experimentally. **C**, Shear modulus depending on fiber concentration. The shear modulus was calculated from the shear stress resulting from a 10% shear strain. **D-F**, Stress relaxation with varying strains and fiber concentrations. All cases exhibited strain-enhanced stress relaxation; stress relaxes faster with a larger strain.

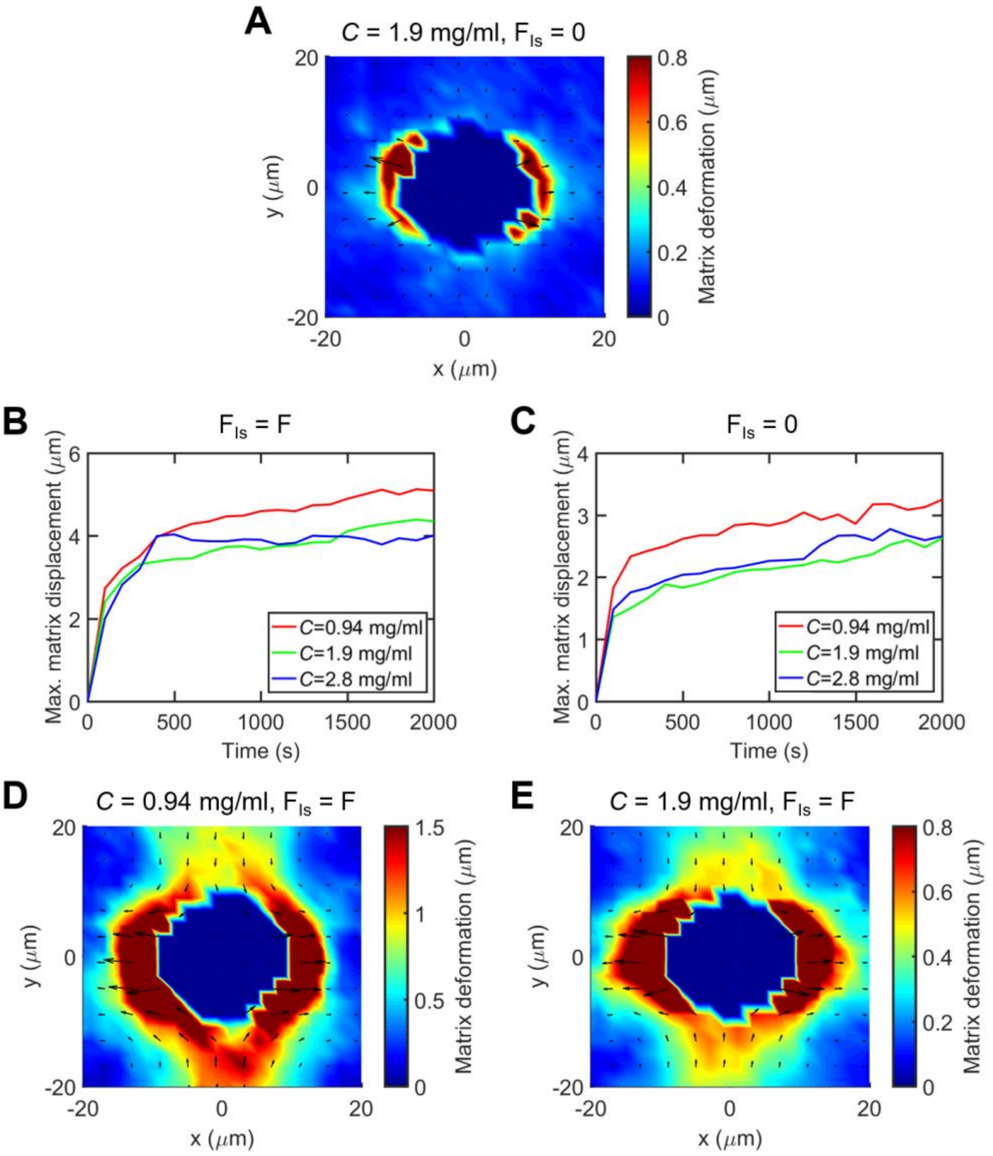

Fig. S13. Incorporating spindle elongation to simulations facilitates cell elongation during division. A, Matrix deformation with 1.9 mg/ml without spindle elongation. Simulation results of maximum matrix displacement **B**, with and **C**, without incorporating spindle elongation. Matrix deformation under the condition of **D**, 0.94 mg/ml with spindle elongation, and **E**, 1.9 mg/ml with spindle elongation.

Supplementary table 1. List of parameters employed in the computational model.

Symbol	Definition	Value
$r_{0,\mathrm{f}}$	Length of fibers	1.0×10^{-6} [m]
$r_{\mathrm{c,f}}$	Diameter of fibers	6.5×10^{-9} [m]
$\theta_{0,\mathrm{f}}$	Bending angle formed by adjacent fibers	0 [rad]
$\kappa_{\mathrm{s,f}}$	Extensional stiffness of fibers	4.0×10^{-3} [N/m]
$\kappa_{\mathrm{b,f}}$	Bending stiffness of fibers	4.14×10^{-19} [N·m]
$r_{0,\mathrm{xl}}$	Length of a cross-linker arm	2.0×10^{-7} [m]
$r_{\mathrm{c,xl}}$	Diameter of a cross-linker arm	1.0×10^{-8} [m]
$\theta_{0,\mathrm{xl},1}$	Bending angle formed by two cross-linker arms	0 [rad]
$\theta_{0,\mathrm{xl},2}$	Bending angle formed by a cross-linker arm and the axis of a fiber where the arm is bound	π/2 [rad]
$\kappa_{\mathrm{s,xl}}$	Extensional stiffness of cross-linkers	2.0×10^{-3} [N/m]
$\kappa_{\mathrm{b,xl},1}$	Bending stiffness 1 of cross-linkers	1.04×10^{-19} [N·m]
$\kappa_{\mathrm{b,xl},2}$	Bending stiffness 2 of cross-linkers	1.04×10^{-19} [N·m]
$r_{0,\mathrm{m}}$	Initial length of membrane elements	4.0×10^{-7} [m]
$\theta_{0,\mathrm{m}}$	Bending angle formed by adjacent membrane elements	0 [rad]
$\kappa_{\mathrm{s,m}}$	Extensional stiffness of membrane	1.0×10^{-4} [N/m]
$\kappa_{\mathrm{b,m}}$	Bending stiffness of membrane	1.0×10^{-18} [N·m]
κ_{r}	Strength of repulsive force	4×10^{-4} [N/m]
κ_{v}	Strength of volume conservation	
C_{f}	Fiber concentration	0.94-2.8 [mg/ml] (=0.4-1.2 [μM])
$\langle L_{\mathrm{f}} \rangle$	Average length of fibers	~9.3-11.6 [μm]
R_{xl}	Molar ratio of cross-linkers to fibers (= cross-linker concentration / fiber concentration)	0.03
N_{m}	Number of membrane elements or nodes	158
Δt	Time step	3.97×10^{-4} [s]
μ	Viscosity of medium	8.6 [Pa·s]
$k_{0,\mathrm{u}}$	Zero-force unbinding rate constant of cross-linkers	1×10^{-6} [s^{-1}] (= $k_{0,\mathrm{u}}^{*}$)
λ_{u}	Force sensitivity of cross-linker unbinding	4.0×10^{-10} [m] (λ_{u}^{*})
$k_{\mathrm{B}}T$	Thermal energy	4.142×10^{-21} [J]

Captions for Videos

Video 1. Cells dividing in collagen gels deform the surrounding collagen fibers as they progress mitosis. Division of single MDA-MB-231 cell in a collagen gel (left) and corresponding matrix deformation of the collagen gel (right). The dividing cell in a collagen gel exerts outward forces and pushes away the surrounding collagen fibers along the mitotic axis. Matrix deformation was visualized by tracking collagen fibers. Scale bar is 10μm.

Video 2. Cells dividing in collagen gels of the low density buckle collagen fibers. The region of the buckled collagen fibers is indicated in Figure 1H. The yellow arrows indicate the onset of buckling. Scale bar is 10μm.

Video 3. Cells dividing in collagen gels of the medium density deform the surrounding collagen fibers during mitosis. Division of a cell in a collagen gel of medium density (left) and corresponding matrix deformation (right). Scale bar is 10μm.

Video 4. Cells dividing in collagen gels of the high density deform the surrounding collagen fibers during mitosis. Division of a cell in a collagen gel of high density (left) and corresponding matrix deformation (right). Scale bar is 10μm.

Video 5. Cells that divide after remodeling collagen network deform the surrounding collagen fiber along the mitotic axis during mitosis. Division of a cell that was cultured in a collagen gel for one day (left) and corresponding matrix deformation (right). Densified collagen

fibers are observed around the cell, indicating that the cell remodeled the surrounding collagen fibers prior to entering mitosis. Scale bar is 10µm.

Video 6. Cells that divide under MMP inhibitors deform the surrounding collagen fiber along the mitotic axis during mitosis. Division of a cell that was cultured in a collagen gel for one day in the presence of MMP inhibitors (left) and corresponding matrix deformation (right). Densified collagen fibers are still observed around the cell under MMP inhibitors, indicating that the cell was able to remodel the surrounding collagen fibers prior to entering mitosis independently of the inhibition. Scale bar is 10µm.

Video 7. Laser ablation performed on spindles. Laser ablation was performed to sever mitotic spindles for cells entering anaphase. Microbeads (yellow) were embedded in collagen gels to visualize relaxation of collagen deformation. The position of microbeads retracts in the direction of the cells shortly after ablation. White line represents the region of laser ablation, and white arrows indicate microbeads that are notably displaced inwardly after ablation. Scale bar is 10µm.

Video 8. Laser ablation performed on part of cell body where spindles were not positioned. Laser ablation was performed for cells entering anaphase on part of cell body where spindles were not positioned along an axis parallel to the interpolar spindle. Microbeads (yellow) were embedded in collagen gels to visualize relaxation of collagen deformation. White line represents the region of laser ablation. Scale bar is 10µm.

Video 9. 3D reconstruction of a dividing cell. Time-lapse images of a dividing cell were three-dimensionally reconstructed. Scale bar is 10µm.
